# Identification and quantification of honeybee venom constituents by multiplatform metabolomics

**DOI:** 10.1038/s41598-020-78740-1

**Published:** 2020-12-10

**Authors:** Agnieszka Klupczynska, Szymon Plewa, Paweł Dereziński, Timothy J. Garrett, Vanessa Y. Rubio, Zenon J. Kokot, Jan Matysiak

**Affiliations:** 1 Department of Inorganic and Analytical Chemistry, Poznan University of Medical Sciences, 60-780 Poznan, Poland; 2 Department of Pathology, Immunology and Laboratory Medicine, College of Medicine, University of Florida, Gainesville, FL 32610 USA; 3 Faculty of Health Sciences, Calisia University – Kalisz, Poland, 62-800 Kalisz, Poland

**Keywords:** Analytical chemistry, Metabolomics, Entomology

## Abstract

Honeybee (*Apis mellifera*) venom (HBV) has been a subject of extensive proteomics research; however, scarce information on its metabolite composition can be found in the literature. The aim of the study was to identify and quantify the metabolites present in HBV. To gain the highest metabolite coverage, three different mass spectrometry (MS)-based methodologies were applied. In the first step, untargeted metabolomics was used, which employed high-resolution, accurate-mass Orbitrap MS. It allowed obtaining a broad overview of HBV metabolic components. Then, two targeted metabolomics approaches, which employed triple quadrupole MS, were applied to quantify metabolites in HBV samples. The untargeted metabolomics not only confirmed the presence of amines, amino acids, carbohydrates, and organic acids in HBV, but also provided information on venom components from other metabolite classes (e.g., nucleosides, alcohols, purine and pyrimidine derivatives). The combination of three MS-based metabolomics platforms facilitated the identification of 214 metabolites in HBV samples, among which 138 were quantified. The obtaining of the wide free amino acid profiles of HBV is one of the project’s achievements. Our study contributed significantly to broadening the knowledge about HBV composition and should be continued to obtain the most comprehensive metabolite profile of HBV.

## Introduction

Honeybee (*Apis mellifera*) venom (HBV) is a natural product that contains a huge variety of bioactive compounds, such as proteins, biogenic amines, carbohydrates, and free amino acids^1,2^. Peptides and proteins constitute the majority of HBV dry mass. The major component of HBV is melittin, a small protein containing 26 amino acid residues, representing 40–60% of the dry venom^3^. This peptide is regarded as the main toxin causing pain and inflammation associated with bee stings^4^; however, it only induces mild allergic reactions^5^. Among other peptide components of HBV, occurring at a lower quantity, are apamin, mast cell-degranulating (MCD) peptide, adolapin, secapin, and procamine^1,2^. HBV also contains a number of enzymes, with phospholipase A2 being the most allergenic and immunogenic protein as well as the second most abundant compound in HBV (10–12% of the dry venom)^6^.

The advent of high-throughput omics technologies (transcriptomics, proteomics, metabolomics) has revolutionized the study of venoms since they facilitate large-scale data collection and analysis. Moreover, omics methodologies, usually employing mass spectrometry, are characterized by high sensitivity and selectivity, and thus enable to identify and characterize low-abundance venom components^7^. HBV has been a subject of extensive proteomics research, which led to the identification of a long list of peptide and protein components^8–11^. However, scarce information on the metabolite composition of HBV can be found in the available literature. Only few classes of compounds are mentioned among metabolites present in HBV: biologically active amines (histamine, dopamine, norepinephrine, serotonin)^12–14^, free amino acids (α-amino acids, β-aminoisobutyric acid)^12,15^, carbohydrates, and lipids^16^. Limited knowledge of metabolite components of HBV results from a limited number of metabolomics studies performed. To date, modern metabolomics platforms have rarely been used in venom studies, including HBV^17^. Recently, we have conducted the determination of a panel of organic acids in HBV using liquid chromatography-tandem mass spectrometry-based method^18^. Nonetheless, there is still a lack of detailed studies on the determination of such significant groups of metabolites as free amino acids or lipids.

Due to incomplete information on HBV composition and the huge potential of using HBV in medicine, there is a need for further omics research of this bee product. The HBV and its major constituents exhibit a wide variety of pharmacological properties, such as anti-inflammatory, analgesic, antiviral, antimicrobial, and anti-cancer^2,19,20^. The therapeutic applications of HBV include i.a. rheumatoid arthritis^21^, musculoskeletal pain^22^, neurodegenerative diseases (Parkinson’s disease, Alzheimer’s disease)^2,23^, skin diseases^24^, and neoplastic diseases^1^. Many in vitro and in vivo studies indicated that HBV has a direct antitumor effect and leads to inhibition of proliferation, metastasis, angiogenesis, and induction of apoptosis^1,25^. Moreover, some promising findings suggested that HBV can be used in combination with standard chemotherapy, e.g., cisplatin^26,27^. Another important application of HBV is venom immunotherapy, which is an effective treatment of severe honeybee sting-induced allergic reactions^28^. We have gained knowledge of the mechanism of action of selected HBV compounds^19,29^. However, many questions remain unanswered, such as synergistic interactions of the venom components, the presence of non-protein allergenic compounds or toxic compounds. With modern metabolomics technologies, such as liquid chromatography-mass spectrometry or gas chromatography-mass spectrometry, comprehensive studies of venoms became achievable^17^. Analysis of metabolite profiles in cell lines or living organisms can also aid in a better understanding of response induced by exposure to HBV and its components^30^.

The aim of the study was to identify and quantify the metabolites present in HBV using mass spectrometry-based metabolomics. Metabolomics approach has already been used to study changes in serum metabolite levels in honeybee sting victims^31^. However, until now, there has been no comprehensive report on HBV metabolome, which remains unexplored. The vast majority of HBV studies are focused on protein and peptide compounds. To gain the highest metabolite coverage, three different mass spectrometry (MS)-based methodologies were applied. In the first step, untargeted metabolomics was used, which employed high-resolution, accurate-mass Orbitrap mass spectrometry. It allowed obtaining a broad overview of HBV metabolic components, however, without quantitative data. Then, targeted metabolomics approaches, which employed triple quadrupole (QqQ) mass spectrometry in combination with chromatographic separation and flow injection analysis, were applied to quantify metabolites in HBV samples.

## Results

### LC-Q-Orbitrap-MS untargeted metabolomics analysis of HBV

The conducted experiments allowed to identify 63 low-molecular-weight compounds in positive ionization mode and 46 compounds in negative ionization mode, with 23 compounds identified in common (Supplementary Table S1). This resulted in a total number of 86 unique metabolites identified in HBV. The research allowed us to identify metabolites belonging to such classes as amino acids, catecholamines, organic acids, carbohydrates, purines, pyridines, pyrimidines, nucleosides, and nucleotides (Table 1). The most common metabolite class found in HBV extracts was amino acids and derivatives. This class comprised nearly 40% of all metabolites found in HBV. HBV solutions contained both proteinogenic and non-proteinogenic amino acids.

**Table 1:** A list of low-molecular-weight compounds identified in honeybee venom solutions in the untargeted metabolomics study.

Metabolite class	Compound name
Alcohols and polyols	Pantothenic acid; quinic acid
Amines	Histamine; phenylethylamine; tyramine
Amino acids, peptides, and analogs	1-Aminocyclopropanecarboxylic acid; 3-aminoisobutanoic acid; 3-O-methyldopa; 4-guanidinobutanoic acid; acetylglycine; alanine/sarcosine; arginine; aspartic acid; betaine; gamma-aminobutyric acid; glutamic acid; glutamine; glycine; isoleucine; l-Dopa (levodopa); leucine; methionine; *N*-alpha-acetylarginine; *N*-alpha-acetyllysine; *N*-acetylglutamic acid; *N*-acetylalanine;* N*-acetylaspartic acid; *N*-methyl aspartic acid; ornithine; phenylalanine; pipecolic acid; proline; pyroglutamic acid (5-oxoproline); serine; taurine; tryptophan; tyrosine; ureidopropionic acid; valine
Carbohydrates and carbohydrate conjugates	Glucose/fructose; gluconic acid; glucosamine; glucuronic acid/galacturonic acid; glyceraldehyde; glyceric acid; tartaric acid; vanilloloside
Catecholamines and derivatives	Dopamine; norepinephrine
Imidazoles	Allantoin; imidazoleacetic acid
Nucleosides, nucleotides, and analogs	Adenosine 5′-monophosphate; cytidine; guanosine; uridine; uridine 5′-monophosphate
Organic acids and derivatives	3-Methyl-2-oxovaleric acid; citric acid; fumaric acid; glycolic acid; isocitric acid; lactic acid; malic acid; malonic acid; *N*-Acetylputrescine; succinic acid
Phenols	3,4 Dihydroxyphenylacetic acid; homovanillic acid
Purines and purine derivatives	Adenine; hypoxanthine; uric acid; xanthine
Pyridines and derivatives	Nicotinic acid; pyridoxal
Pyrimidines and pyrimidine derivatives	5-Methylcytosine; cytosine; dihydrouracil; thymine; uracil
Tryptamines and derivatives	Serotonin; tryptophanamide
Other metabolites	3-(2-Hydroxyphenyl)propanoic acid; carnitine; citramalic acid; hippuric acid; kynurenic acid; (+)-salsolinol

Among the identified compounds, some isomers and isobars occurred, e.g., citric acid and isocitric acid, isoleucine and leucine, taurine, and 5-methylcytosine (Supplementary Table S1). Chromatographic separation was achieved in those cases. The only exception was alanine and its isomers. Improved chromatographic separation was applied in the targeted metabolomics approach, which allowed to obtain separate signals for alanine, β-alanine, and sarcosine.

Among the identified low-molecular-weight compounds, one non-endogenous compound was found in HBV. The ion at m/z = 152.0161 was identified as a proton adduct of benzisothiazolinone. This xenobiotic is a commonly used biocide with microbicide and fungicide mode of action, which induces occupational allergic contact dermatitis and airway inflammatory diseases^32,33^. Benzisothiazolinone was detected in only one HBV extract. However, this indicated the need to monitor pesticide residues in honeybee products to assess their potential risk to human health and provide information on the pesticide treatments for field crops surrounding the apiaries. Crops treated with pesticides are known to be highly toxic to pollinators and several studies have indicated a link between bee deaths and pesticide use^34^.

### LC–MS targeted metabolomics analysis of HBV

Within the project, two targeted metabolomics experiments were performed. First, wide-spectrum metabolite determination was conducted, which covered such metabolite classes as amino acids, biogenic amines, acylcarnitines, glycerophospholipids, and sphingolipids (Supplementary Table S2). It revealed that among the most abundant and diverse metabolite classes quantified in the HBV samples were amino acids and their derivatives. For this reason, the amino acid determination in HBV was expanded using another LC–MS/MS-based methodology. The second targeted metabolomics experiment allowed for the quantification of a broad spectrum of amino acids (both proteinogenic and non-proteinogenic) and amino acid derivatives (Supplementary Table S3). Despite the applied two targeted metabolomics strategies have some common analytes (mainly in the group of proteinogenic amino acids), the second methodology allowed for quantitation of additional 17 metabolites in HBV, most of them for the first time (Supplementary Fig. S1). The first targeted method lacks γ-aminobutyric acid (GABA) and β-alanine among analytes, which are essential venom constituents known from the literature^35^. However, the method provides the opportunity to determine a broad spectrum of lipid metabolites in HBV samples (Supplementary Table S2). It can be concluded that the two applied targeted methodologies complement each other and were equally useful in the performed HBV metabolomics research.

#### Wide-spectrum metabolite determination using AbsoluteIDQ p180 kit and LC-QqQ-MS/MS

Three pooled HBV extracts were analyzed using the AbsoluteIDQ p180 kit: methanolic extract, aqueous extract with 0.1% formic acid, and DMSO extract. Unsurprisingly, the obtained chemical profiles of the HBV depend on the extraction solvent used. Overall, 145 compounds were identified in HBV: 22 acylcarnitines, 40 amino acids and amines, 56 phosphatidylcholines, 11 lysophosphatidylcholines, 15 sphingomyelins, and a sum of hexoses (Supplementary Table S2). Of the 145 metabolites identified, 108 were found in the methanolic extract, 74 were found in the aqueous extract, and 138 in the DMSO extract of HBV. DMSO extract of HBV allowed us to determine the largest number of glycerophospholipids. The levels of amino acids were generally the highest in HBV extract prepared in 0.1% formic acid in water, whereas the levels of acylcarnitines were the highest in the methanolic extract.

Some of the detected metabolites exhibited concentration levels either above the upper limit of quantification (ULOQ) or below the lower limit of quantification (LLOQ) (Supplementary Table S2). Therefore, 127 out of 145 compounds were quantified in the venom samples. Too high levels (exceeding ULOQ) of proline, dopamine, histamine, serotonin, and putrescine were observed in all studied HBV extracts. In turn, very low concentrations of acylcarnitines were revealed in HBV.

**Figure 1:**
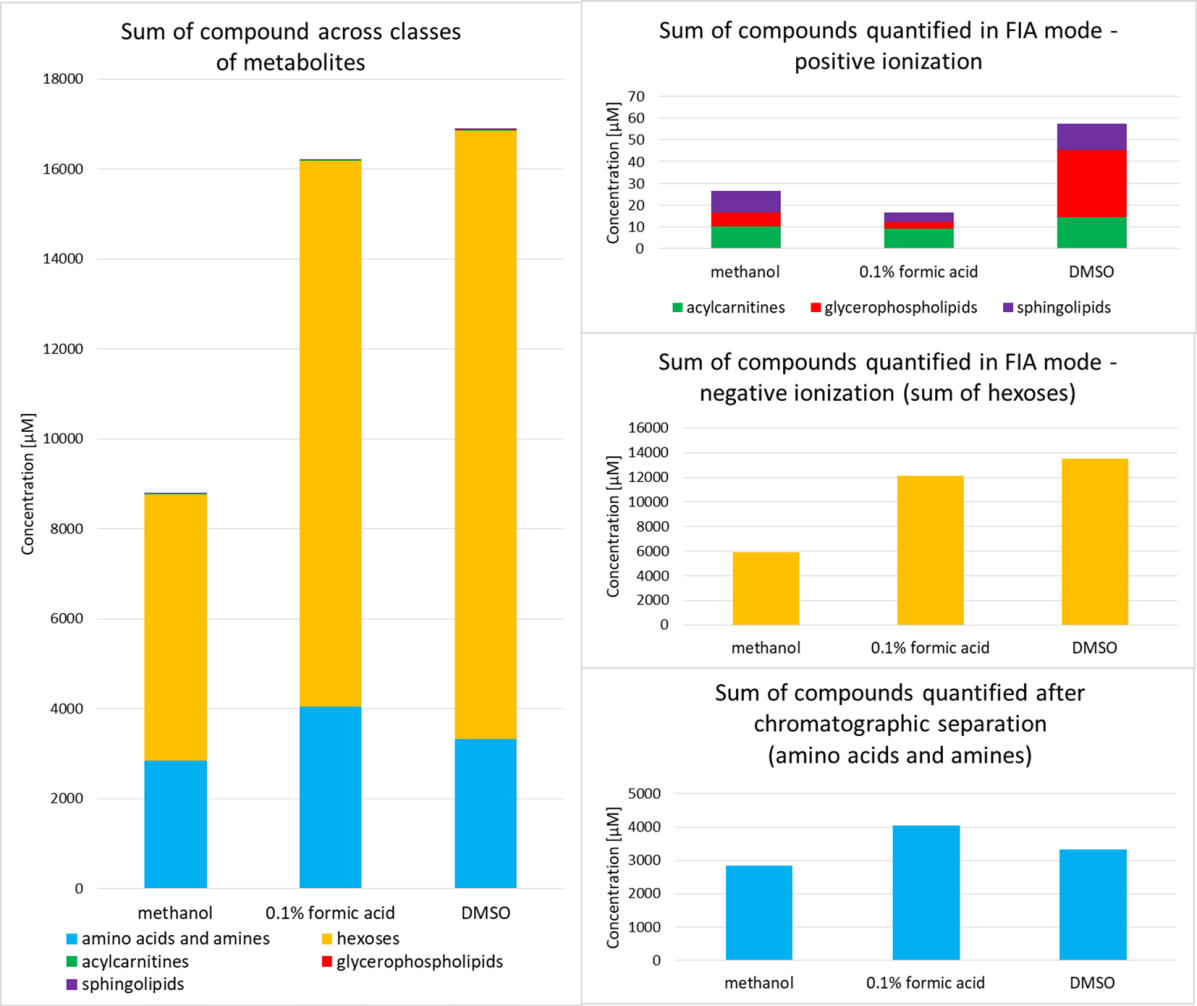
Comparison of cumulative metabolite profiles of different honeybee venom extracts. Three different solvents were used for the venom sample preparation: 0.1% formic acid in water, methanol, and dimethyl sulfoxide (DMSO). Bars are color-coded by metabolite class.

The study showed that the determined metabolite profile of HBV extract consisted mainly of monosaccharides, amino acids, and biogenic amines (Fig. 1). Lipid constituents (acylcarnitines, sphingomyelins, and glycerophospholipids) are a minority of the determined HBV profile. Although nearly 100 lipid metabolites were determined in HBV samples, they constituted only up to 0.007% of the dry mass of the venom. The most abundant HBV components in each assayed metabolite class were: dopamine, histamine, putrescine, serotonin, spermidine among amines, proline, glycine, alanine, lysine, taurine, arginine, glutamic acid, aspartic acid among amino acids, carnitine, hydroxyhexadecenoylcarnitine, dodecenoylcarnitine among acylcarnitines, PC aa C34:2, PC aa C36:2, PC aa C34:1, PC aa C36:4 among glycerophospholipids, and SM C18:0, SM C16:0 among sphingolipids (Supplementary Table S2). No previous study has evaluated levels of acylcarnitines, glycerophospholipids, and sphingolipids in HBV extracts.

#### Wide-spectrum amino acid determination using aTRAQ kit and LC-QqQ-MS/MS

In the first step, a pilot study aimed at the optimization of HBV sample treatment was conducted using a pooled venom sample. Supplementary Figure S2shows a workflow applied to select the most appropriate solvent for the determination of free amino acids in HBV. The obtained results clearly demonstrate that a mixture of methanol:DMSO:0.1% formic acid (1:1:1, *v*/*v*/*v*) was the best choice for HBV dissolving prior to amino acid determination using the aTRAQ methodology (Supplementary Fig. S3).

**Figure 2:**
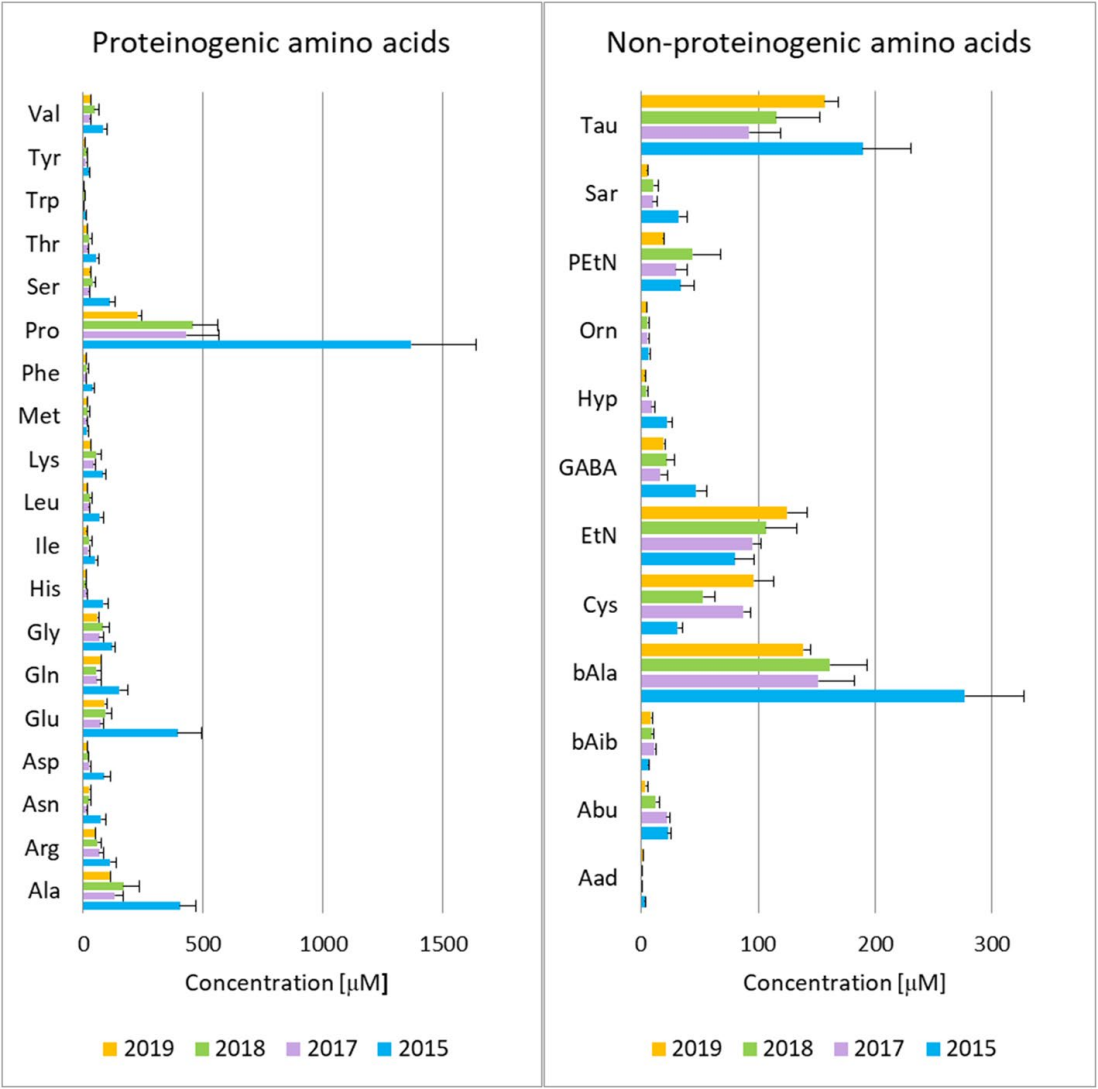
Free amino acid profiles determined in honeybee venom samples. Amino acid profiles (average and SEM) determined in solutions (c = 50 mg/ml) of honeybee venom samples collected in different years. Amino acid abbreviations are defined in Supplementary Table S3.

The applied method enabled quantitative analysis of both proteinogenic and non-proteinogenic amino acids in HBV. The full list of analyzed compounds is contained in Supplementary Table S3. 31 out of 42 amino acids were determined in each HBV extract and the remaining 11 amino acids occurred in the analyzed samples below the lower level of quantification. Figure 2 displays the determined amino acid profiles as well as differences in amino acid content between HBV samples collected in different years. The obtained amino acid profiles of HBV differed only quantitatively, not qualitatively. To further assess the differences in amino acid composition between the studied HBV samples, principal component analysis (PCA) was performed. The obtained score plot showed a grouping of HBV samples according to the assignment of the sample to the year of sampling (Supplementary Fig. S4.A). What is interesting, partial clustering was also observed when the grouping variable was a month of sample collection (Supplementary Fig. S4.B). The differences in amino acid profiles between HBV samples collected within the same year, but in various months were depicted in more detail in Fig. S5.

**Figure 3:**
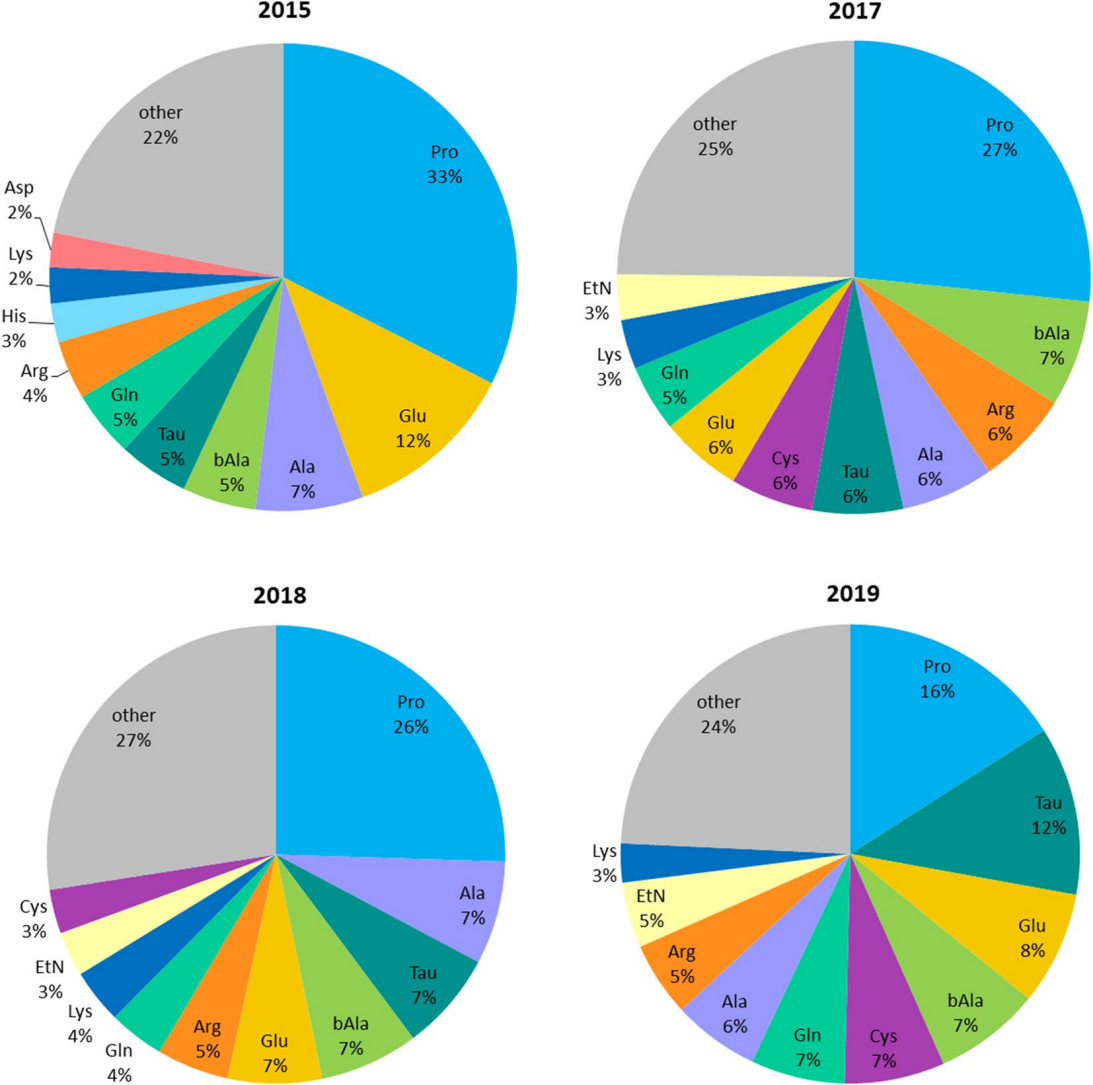
A pie chart showing amino acids determined in the highest amount in honeybee venom samples collected in different years. The study demonstrated that proline is the dominant amino acid constituent of the venom and its content increases with the storage time of the samples. Abbreviations: *Ala* alanine, *Arg* arginine, *Asp* aspartic acid, *bAla* β-alanine, *Cys* cystine, *EtN* ethanolamine, *Gln* glutamine, *Glu* glutamic acid, *His* histidine, *Lys* lysine, *Pro* proline, *Tau*, taurine.

Despite the observed variations in the levels of the studied amino acids, proline was clearly the most abundant amino acid in each analyzed venom sample (Fig. 2). The percent content of this amino acid in a total amino acid profile was higher in older venom samples (Fig. 3). Apart from proline, the most abundant proteinogenic amino acids quantified in HBV were: glutamic acid, alanine, arginine, glutamine, lysine, and glycine (Fig. 3, Supplementary Table S4). These findings are in line with the results of the performed wide-spectrum metabolite determination in HBV. Among non-proteinogenic amino acids and amino acid derivatives, taurine, β-alanine, cystine, ethanolamine, phosphoethanolamine, and GABA constituted the highest percentage of the dry weight of HBV (Fig. 3, Supplementary Table S4).

## Discussion

In the current study, we used three different MS-based methodologies for reliable and comprehensive metabolite detection and quantification in HBV samples. This has been the first study using global metabolomics profiling in bee venom analysis. The performed untargeted experiments showed that HBV has a more complex composition than reported in the literature. The study identified several dozen metabolites present in bee venom (Table 1). The untargeted metabolomics not only confirmed the presence of amines, amino acids, carbohydrates, and organic acids in HBV but also provided information on venom components from other metabolite classes previously undetected in HBV, such as nucleosides, ribonucleotides, alcohols, purine derivatives, and pyrimidine derivatives. The performed global profiling indicated an abundance and variety of amino acids and amino acid derivatives in HBV. Therefore, we paid special attention to this metabolite class in the subsequent targeted metabolomics studies of HBV.

The applied two targeted methodologies (AbsoluteIDQ p180 and aTRAQ) have already been used in metabolomics research of cancer^36,37^, cardiometabolic diseases^38,39^, neuroscience^40^, pharmacology and toxicology^41^. It is the first time that such methods have been applied in venom analysis. Therefore, various solvents were tested for the preparation of HBV solution. These optimization steps showed that the solvent used to extract metabolites from HBV plays an important role (Fig. 1, Supplementary Fig. S3). In the case of the AbsoluteIDQ p180 kit, it was hard to select the most appropriate solvent since the different physicochemical properties of the assayed compounds. Thus, we showed HBV metabolite profiles depending on the solvent used (Fig. 1, Supplementary Table S2). It can be concluded that different solvents should be used to detect and determine in HBV as many compounds as possible and thus obtain complementary results.

The application of targeted metabolomics methodologies led to the identification of 156 metabolites in HBV samples. Among them, 138 were quantified. The remaining compounds yielded too low or too high signals, which resulted in concentration values below LLOQ or above ULOQ of the method. Quantitative analysis of venom metabolome presents a great analytical challenge not only due to the tremendous chemical diversity of metabolites but also due to the fact that the concentrations of individual metabolites vary dramatically. Villar-Briones et al.^42^ analyzed organic and peptidyl components of snake venoms and reported that organic acid abundances observed in the assayed samples span nearly eight orders of magnitude. Similarly, polyamine levels in snake venoms span several orders of magnitude^43^. In our investigation of HBV, amines reached mostly values exceeding the method ULOQ. However, data on the content of biogenic amines in HBV have already been known from the literature^13,14^. The most abundant amines occurring in HBV are dopamine, histamine, and serotonin, which is consistent with our study. The current paper filled the gap regarding the content of metabolites from other classes in HBV, such as amino acids, acylcarnitines, and glycerophospholipids.

The performed study allowed the determination of concentrations of a diverse range of metabolites in HBV samples covering both lipid and polar compounds. The range of the measured metabolites lacked organic acids, which is an important metabolite class present in animal venoms^42^. However, the profiles of low-molecular-weight organic acids have already been studied by our team^18^. The study demonstrated that citric acid was the most abundant acid in HBV and constituted at least 7.3% of the venom dry weight.

Although some differences in metabolite profiles between various HBV extracts and the two applied targeted methods, some common features of HBV metabolome can be observed. The study showed that amines, amino acids and amino acid derivatives were among the major low-molecular-weight components of bee venom. Our research provided specific data on profiles of metabolites from these classes. Till now, limited data on amino acid levels in HBV can be found in the available literature. Therefore, many review papers provide no detailed list of amino acids occurring in this honeybee product^1,2,6^. A partial quantitative analysis of free amino acid fraction was performed by Nelson and O’Connor^15^, who determined 19 amino acids in *Apis mellifera* venom. Surendra et al.^44^ quantified eight amino acids in venom reservoir extract of three Indian honeybee species (*Apis cerana*, *Apis dorsata*, and *Apis florea*). The current artic presents the broadest amino acid profile of *Apis mellifera* venom that has been obtained so far. The application of two targeted metabolomics methodologies allowed us to determine 35 amino acids and their derivatives in HBV. The free amino acid profiling conducted using HBV samples collected in different years allowed for the observation that many amino acids reached the highest concentrations in the oldest venom samples. This trend could be associated with protein degradation during long-term storage and it was most pronounced in the case of proline (Fig. 3). The percent content of proline increases with the storage time, and therefore, proline could be regarded as a marker of the storage stability of venom samples. It should be noted that “proline index”(free proline to free amino acid ratio) is recommended as a marker of freshness and nutritional value of another bee product—bee pollen^45–47^. Chemical variability of bee venom, which results in composition inconsistency, poses a limiting factor in the development of venom-derived medicines. Methods that can be potentially used for bee venom standardization have been recently reviewed^48^. Our study demonstrated that metabolomics research can lead to propose new strategies for HBV standardization that will utilize low-molecular-weight components, such as proline.

The performed research demonstrated that HBV contains a range of physiologically active amines, such as histamine, norepinephrine, dopamine, serotonin. Monoamines are important constituents of many animal venoms, including spiders, snakes, scorpions, insects^49–52^. These metabolites act in the central nervous system as neuromodulators but also have their own functions in the periphery. The most common function of histamine is inflicting pain at the site of venom injection to deter a predator and in defense. Moreover, this amine causes vasodilatation and induces vascular hyperpermeability, which facilitates access of venom components into the blood circulation of prey^12,53^. Dopamine and norepinephrine increase heart rate, and thus they also enhance the distribution of other fractions of bee venom to their sites of action^54^. Catecholamines in *Hymenoptera* venom may also prolong the local action of the venom by constricting capillaries and venules^55^. In hymenopterans, the venom is mainly used as a defense against other insects. Therefore, the physiological effect of dopamine on the acceleration of the hemolymph circulation was proved^56^. Serotonin (5-hydroxytryptamine) contributes to the pain caused by the venom, causes lethal vasoconstriction in smaller predators as well as neurotoxicity in insects^14,57^. A study involving the injection of HBV into one hind paw of a rat indicated a facilitating role for 5-hydroxytryptamine 1A receptor in bee venom-induced inflammatory pain^58^.

The conducted research also provided data on polyamines occurring in HBV. Among them, putrescine and spermidine were present in the highest abundance (Supplementary Table S2). Putrescine is a short-chain aliphatic diamine that can influence the biological activity of histamine. Putrescine inhibits histamine catabolism, what potentiates the histamine toxicity^59,60^. In addition, the anti-aggregating effect of putrescine and spermidine was proved on platelets of normal and hypercholesterolemic rabbits^61^. Putrescine, spermidine, and spermine were also found to disrupt blood–brain barrier integrity^62^. A study of polyamine profiles (putrescine, spermidine, cadaverine, and spermine) was conducted in 31 pooled snake venoms and showed that quantities of each polyamine vary dramatically among taxa. Despite high abundancies of polyamines in snake venoms, their ability to trigger a systemic response in the prey is unclear and local effects seem more probable. Spermine and putrescine showed the highest levels and the greatest dispersion of values in the studied samples^43^. In contrast to snake venom, spermine is an extremely minor constituent in HBV, and spermidine seems to be a more significant component.

The performed metabolomics study enabled the detection and quantification of a wide range of amino acids and their derivatives in HBV. The obtained amino acid profile of HBV covers amino acid neurotransmitters. The high content of inhibitory neurotransmitters, such as glycine, β-alanine, taurine, GABA, was found in the samples. It is thought that these neuroactive amino acids may promote paralysis of insect prey^63^. The inhibitory effect of the mentioned amino acids is more pronounced in insects since their open circulatory system has direct contact with all internal organs. In mammals, amino acids from HBV are quickly metabolized in the liver. Apart from inhibitory neurotransmitters, excitatory transmitters (glutamic acid and aspartic acid) were also determined in HBV. Glutamic acid is recognized as the principal excitatory neurotransmitter in the central nervous system of vertebrates. However, in insects, this amino acid acts both as an excitatory and inhibitory transmitter and plays a major signaling role within their central nervous system^64^. Glutamic acid is also the main excitatory neurotransmitter at the insect neuromuscular junctions, being a counterpart of acetylcholine at mammalian neuromuscular junctions^65–67^. Our findings are in line with Abe et al.^63^, who analyzed amino acid composition in extracts from hornet venom sacs and detected large quantities of neuroactive amino acids. Moore et al.^35^ showed that venom from *Ampulex compressa*, a solitary parasitoid wasp, contains not only high levels of GABA but also comparable concentrations of the GABA receptor agonists taurine and β-alanine. The authors demonstrated that GABAergic chloride channel activation causes central synaptic block and transient paralysis of a cockroach host. In the study of HBV, we found comparable amounts of taurine and β-alanine, but GABA occurs at a much lower level (Supplementary Table S4).

Our study revealed a high abundance of proline in HBV. Proline is a major component of peptide components of HBV, which may explain the presence of this amino acid in the venom. Jamasbi et al.^68^ indicated that proline residue plays a key role in antimicrobial activity and cytotoxicity of melittin, the main constituent of HBV. The proline residue is also found in other antimicrobial peptides derived from HBV, such as apamin, melectin, and secapin^69,70^. The latter one contains a large proportion of proline comprising 5 proline residues. Proline-rich peptides represent an important group of insect antimicrobial peptides^71^. Moreover, proline is the main constituent of hypotensive peptides (bradykinin potentiating peptides) occurring in snake venoms^72^. Villar-Briones and Aird^42^ reported that proline and arginine are the most abundant free amino acids in snake venoms (Elapidae, Viperinae, and Crotalinae). Our research also showed high content of arginine in HBV (Fig. 3).

Metabolites found in HBV samples exhibit an impressive catalog of functions. Among them, neurotransmitters and neurotransmitter precursors represent an important group that requires further attention. The presence of this group of metabolites is believed, in part, to be responsible for the neurotherapeutic effects of HBV. One of the promising applications of HBV is Parkinson’s disease^29^. HBV is a mixture of neuroactive molecules and the observed beneficial effects in the treatment of Parkinson’s disease may result from synergic action of its components, including apamin, melittin, phospholipase A2, and dopamine^73–75^. What is interesting, the current paper shows that HBV contains not only dopamine but also L-Dopa, a precursor of dopamine (Table 1).

The study shows the power of the combined metabolomics strategies. The application of three LC–MS-based metabolomics platforms, demonstrated in the current article, added significantly to broadening the knowledge of HBV composition and brought a substantial contribution to the development of animal venom metabolomics. Taking into account the use of HBV in medicine, knowing its exact composition is very important. Our extensive analysis of HBV facilitated the identification of 214 metabolites in HBV samples, among which 138 were quantified. The obtaining of the broad free amino acid profiles of HBV is one of the project’s achievements. However, due to the huge chemical diversity of metabolites, no single instrument is sufficient to characterize venom metabolome fully and different analytical platforms should be employed, e.g., a combination of LC–MS and GC–MS or CE–MS^17^. Therefore, metabolomics studies should be continued to obtain the most comprehensive metabolite profile of HBV. Another future direction of HBV metabolomics research should be a comparison of metabolic profiles of venom samples collected in different ways. Proteome and phosphoproteome analyses of HBV indicated that venom collected using electrical stimulation is different from venom extracted manually^11^. In the current study, we used venom collected by electrical stimulation and the impact of the bee venom collection method on the obtained metabolome profiles requires future research.

## Methods

### Venom samples

HBV samples (*Apis mellifera carnica*) were collected in an apiary of the Department of Inorganic and Analytical Chemistry, Poznan University of Medical Sciences, by the electrical stimulation method described by Matysiak et al.^8^. Venom was collected for 2 h during a full activity of bees, which allowed obtaining the highest efficiency of HBV production. After drying, the samples were kept at − 80 °C in darkness and dissolved directly before analysis. To avoid possible bias and batch effect, in all experiments (untargeted and targeted) HBV samples were prepared and analyzed in random order on the same day as one batch. At the moment of use, dried HBV samples were dissolved in an appropriate solvent, vortexed (10 min), and sonicated (10 min). In an untargeted metabolomics experiment, seventeen samples collected in 2015 (from June to August) were used and analyzed in this year. In targeted analyses employing aTRAQ kit, samples collected in 2015 (n = 6), 2017 (n = 3), 2018 (n = 5), and 2019 (n = 2) were used. Those analyses were performed in 2019. In targeted analyses employing the AbsoluteIDQ p180 kit, a pooled sample of venoms collected in the year of analysis (2018) was used.

### LC-Q-Orbitrap-MS untargeted metabolomics

For the untargeted MS-based metabolomics study, HBV samples were dissolved in water to obtain 50 mg/mL solution. 100 μL of the solution was mixed with 20 μL of a mixture of internal standards (creatine-d3, l-leucine-d10, l-tryptophan-2,3,3-d3, salicylic acid-d4, succinic-2,2,3,3-d4 acid). Next, 800 μL of acetonitrile:methanol:acetone (8:1:1, *v*/*v*/*v*) mixture was added for protein precipitation, followed by incubation (4 °C, 30 min). Then, the samples were centrifuged (20,000 rcf, 10 min, 8 °C). The supernatant was transferred to a clean microcentrifuge tube and dried under gentle nitrogen flow (MULTIVAP nitrogen evaporator, Organomation Associates, Inc., Berlin, MA, USA). The dry residue was reconstituted in 100 μL of 0.1% formic acid followed by incubation on an ice bath (15 min). The solution was then centrifuged (20,000 rcf, 10 min, 8 °C), and the supernatant was transferred to a vial.

Untargeted metabolite profiling was conducted using a high-resolution, accurate-mass Q Exactive Hybrid Quadrupole-Orbitrap mass spectrometer (Thermo Fisher Scientific, San Jose, CA, USA) equipped with an electrospray ionization source (ESI) coupled to an ultra-high performance liquid chromatograph UltiMate 3000 (Dionex Corporation, Sunnyvale, CA, USA). An ACE Excel 2 C18-PFP (2.1 × 100 mm, 2.0 μm, Advanced Chromatography Technologies, Aberdeen, Scotland) chromatographic column was used. The mobile phase comprised 0.1% formic acid in water (eluent A) and acetonitrile (eluent B). The further detailed chromatography parameters were listed in Supplementary Table S5. The mass spectrometer operated in full MS mode at a mass range of 70–1000 m/z in positive and negative ionization mode. Next, a pooled sample (a mixture of aliquots of 17 venom extracts) was analyzed in data-dependent MS/MS mode to acquire fragmentation spectra of the most intensive signals (top 5). The remaining mass spectrometry settings were summarized in Supplementary Table S6.

Positive and negative raw data files were independently processed with the MZmine 2.19 software^76^. The resulting peak list contained 6141 and 2984 features in positive and negative ionization modes, respectively. The in-house library of standards, analyzed previously using the same instrument and the same method, was the main tool for metabolite identification. Additionally, Human Metabolome Database^77^ and a mass spectral database mzCloud (https://www.mzcloud.org/) were used. Identification of HBV components was performed based on precursor m/z, fragmentation spectra (in case of online databases), and retention time (in case of the in-house libraries).

### LC–MS targeted metabolomics

#### Wide-spectrum metabolite determination using AbsoluteIDQ p180 kit and LC-QqQ-MS/MS

Metabolite determination in HBV was performed using the AbsoluteIDQ p180 kit (Biocrates Life Sciences AG, Innsbruck, Austria). The methodology allows for simultaneous quantification of 188 metabolites, including acylcarnitines, amino acids, biogenic amines, hexoses, glycerophospholipids and sphingolipids (Supplementary Table S2). This was the first study employing the AbsoluteIDQ p180 kit in animal venom analysis. Therefore, different solvents were used for HBV sample preparation: 0.1% formic acid in water, methanol, and dimethyl sulfoxide (DMSO). Each HBV solution (c = 500 mg/mL) was analyzed in triplicate.

Analyses were conducted on a 96-well plate according to the manufacturer’s recommended protocol. In the first step, a mixture of internal standards (10 µL) was pipetted into the plate. Then, solution of pooled HBV (30 µL), quality control (QC) samples (10 µL), calibrators (10 µL), and zero sample (phosphate buffer saline, 10 µL) were put into appropriate wells. The plate was dried (30 min) under nitrogen flow using a pressure manifold (Positive Pressure Manifold 96 Processor, Agilent Technologies, Santa Clara, CA, USA). In the next step, derivatization using 5% phenylisothiocyanate was performed (20 min). The derivatization was followed by drying under nitrogen flow (60 min). Then, the extraction solvent was added, and the plate was shaken (30 min, 450 rpm). After that, the extract was pushed through the filter layer into a capture plate using nitrogen flow. 150µL of the final extract was transferred into the second 96-well plate intended to LC–MS analysis. The extracts for LC–MS and FIA-MS analyses were diluted using 150 µL of water and 400 µL of the FIA mobile phase, respectively. Both plates were covered by silicone mates, shaked (2 min, 600 rpm), and placed into thermostatic autosampler (10 °C).

The measurements were performed using electrospray ionization triple quadrupole tandem mass spectrometer 4000 QTRAP (Sciex, Framingham, MA, USA) coupled to 1260 Infinity (Agilent Technologies, Santa Clara, CA, USA) high-performance liquid chromatograph. The assays of amino acids and biogenic amines were preceded by chromatographic separation using gradient elution with 0.2% formic acid in water (eluent A) and 0.2% formic acid in acetonitrile (eluent B). The ZORBAX Eclipse XDB-C18 (3.0 × 100 mm, 3.5 μm) column (Agilent Technologies, Santa Clara, CA, USA), with a SecurityGuard pre-column (C18, 4.0 × 3.0 mm, Phenomenex, Torrance, CA, USA) were used. The further detailed chromatography parameters were listed in Supplementary Table S7. The mass spectrometer was operated in MRM (multiple reaction monitoring) mode in positive and negative ionization mode. The detailed mass spectrometry settings were summarized in Supplementary Table S8. Data was acquired using Analyst 1.6 software (Sciex, Framingham, MA, USA) and processed using Biocrates MetIDQ software (Biocrates Life Sciences AG, Innsbruck, Austria).

The methodology was fully validated in line with European Medicine Agency Guideline on bioanalytical method validation and its interlaboratory reproducibility was also proved^78^. Moreover, an automated technical validation was performed using MetIDQ software for each analyzed kit plate to confirm the validity of the run. The method was successfully applied by us earlier in the analysis of other biological matrices^79^.

#### Wide-spectrum amino acid determination using aTRAQ kit and LC-QqQ-MS/MS

Determination of free amino acids in HBV was performed using an aTRAQ kit (Sciex, Framingham, MA, USA) and a fully validated, highly selective liquid chromatography-tandem mass spectrometry (LC–MS/MS) method^31,80^. The applied methodology allows for simultaneous quantitative analysis of 42 analytes, including proteinogenic amino acids, non-proteinogenic amino acids, and their derivatives (Supplementary Table S3). First, a series of analyses was conducted to select the most appropriate solvent for the preparation of HBV solution. Six solvents (water, 0.1% formic acid in water, methanol, ethanol, acetonitrile, and dimethyl sulfoxide (DMSO)) and their mixtures were tested (Supplementary Fig. S2). Then, the solvent yielding the best method performance and signal intensities was chosen for the preparation of 16 HBV solutions.

Forty microliters of HBV solution (c = 50 mg/mL) in an appropriate solvent were used for the analysis. In the first step, 10 μL of sulfosalicylic acid was added to precipitate proteins, followed by vortexing and centrifugation (10,000 rpm, 2 min). Then, 10 μL of supernatant was transferred to a clean tube and mixed with 40 μL of labeling buffer. 10 μL of the obtained mixture was transferred to a clean tube, where amino acid labeling was performed using an aTRAQ Reagent Δ8 solution (5 μL). After vortexing and centrifugation, the vial was incubated (30 min, room temperature). The labeling reaction was stopped by adding 5 μL of hydroxylamine. After incubation (15 min, room temperature), 32 μL of the internal standard solution was added, and the sample was evaporated (15 min, 50 °C) in a vacuum concentrator (miVac Duo Concentrator, Genevac, Stone Ridge, NY, USA). After concentration, the sample was diluted with 20 μL of water and then transferred to an autosampler vial.

Separation and quantitation of the amino acids was performed using a 4000 QTRAP mass spectrometer (Sciex, Framingham, MA, USA) combined with an Agilent Infinity 1260 high-performance liquid chromatograph (Agilent Technologies, Santa Clara, CA, USA). A C18 column (4.6 × 150 mm, 5 μm, Sciex, Framingham, MA, USA) was applied. The mobile phase consisted of 0.1% formic acid and 0.01% heptafluorobutyric acid in water (eluent A) and 0.1% formic acid and 0.01% heptafluorobutyric acid in methanol (eluent B). The chromatographic conditions were provided in detail in Supplementary Table S9. The ESI source worked in a positive ionization mode. The applied ion source settings were provided in Supplementary Table S10. Due to a large number of analytes, a scheduled multiple reaction monitoring (sMRM) mode was used. The list of MRM transitions for each amino acid and its corresponding internal standard, along with collision energies, is contained in Supplementary Table S3. Data acquisition and processing were conducted using Analyst 1.6 software (Sciex, Framingham, MA, USA).

The method validation proved its high specificity, sensitivity, accuracy, and precision^31^. Additionally, 2 QC amino acids (norleucine and norvaline) were used to assess recovery and labeling efficiency in each HBV sample. Before the sequence, the system suitability test was also performed to verify the overall LC–MS/MS performance.

## Supplementary Information

Supplementary Information.
