# Supplementary material for: Identification and quantification of honeybee venom constituents by multiplatform metabolomics
Source: Sci Rep. 2020 Dec 10;10:21645. doi: 10.1038/s41598-020-78740-1 (PMC7729905; doi:10.1038/s41598-020-78740-1)
Supplement: Supplementary file 1 — Supplementary Information. [file 41598_2020_78740_MOESM1_ESM.pdf]

Supplementary information

Identification and quantification of honeybee venom constituents by multiplatform metabolomics

Agnieszka Klupczynska [1,]*, Szymon Plewa [1], Paweł Dereziński [1], Timothy J. Garrett [2], Vanessa Y. Rubio [2], Zenon J. Kokot [3] and Jan Matysiak [1]

[1] Department of Inorganic and Analytical Chemistry, Poznan University of Medical Sciences, 60 - 780 Poznan, Poland
[2] Department of Pathology, Immunology and Laboratory Medicine, College of Medicine, University of Florida, Gainesville, FL 32610, USA
[3] Faculty of Health Sciences, Calisia University – Kalisz, Poland, 62-800 Kalisz, Poland
* Correspondence: aklupczynska@ump.edu.pl

Contents

Supplementary Table S1. Metabolites identified in honeybee venom extracts using LC-MS-based untargeted methodology (arranged alphabetically).

Supplementary Table S2. A list of metabolites determined using AbsoluteIDQ p180 kit in different honeybee venom extracts (methanolic; 0.1% formic acid in water; DMSO).

Supplementary Table S3. A list of MRM transitions for 42 amino acids and their corresponding internal standards along with collision energy values.

Supplementary Table S4. The percentage content of the amino acids quantified in the studied HBV samples collected in different years (percentage of the dry weight of HBV).

Supplementary Table S5. LC parameters for untargeted metabolomics.

Supplementary Table S6. MS parameters for untargeted metabolomics.

Supplementary Table S7. LC parameters for wide-spectrum metabolite determination using AbsoluteIDQ p180 kit.

Supplementary Table S8. MS parameters for wide-spectrum metabolite determination using AbsoluteIDQ p180 kit.

Supplementary Table S9. LC parameters for wide-spectrum amino acid determination using aTRAQ kit.

Supplementary Table S10. MS parameters for wide-spectrum amino acid determination using aTRAQ kit.

Supplementary Figure S1. Venn diagram presenting common and unique analytes covered by the two applied targeted metabolomics methodologies (AbsoluteIDQ p180 kit and aTRAQ kit) among non-proteinogenic amino acids and amino acids derivatives. Both kits enable analysis of the same set of proteinogenic amino acids.

Supplementary Figure S2. A workflow applied to select the most appropriate solvent for determination of free amino acids in honeybee venom samples.

Supplementary Figure S3. Bar chart showing the impact of solvent on the total amino acid concentration (average ± SD) quantified in honeybee venom sample.

Supplementary Figure S4. Scores plots between the first and second principal components (with explained variances shown in brackets) obtained in principal component analysis (PCA) of amino acid profiles in honeybee venom.

Supplementary Figure S5. Amino acid profiles determined in solutions of honeybee venom (c=50 mg/ml) collected in different months and years (A – 2015, B- 2018).

Supplementary Table S1. Metabolites identified in honeybee venom extracts using LC-MS-based untargeted methodology (arranged alphabetically).

Compound name	Metabolite class	Molecular formula	Molecular weight, Da	RT, min	Polarity
(+)-salsolinol	tetrahydroisoquinolines	C10H13NO2	179.2157	4.87	pos
1-aminocyclopropanecarboxylic acid	amino acids, peptides, and analogues	C4H7NO2	101.1039	0.81	pos
3-(2-hydroxyphenyl)propanoic acid	phenylpropanoic acids	C9H10O3	166.1739	8.61	neg
3,4 dihydroxyphenylacetic acid	phenols	C8H8O4	168.1467	6.85	neg
3-aminoisobutanoic acid	amino acids, peptides, and analogues	C4H9NO2	103.1198	0.94	pos
3-methyl-2-oxovaleric acid	organic acids and derivatives	C6H10O3	130.1418	6.73	neg
3-O-methyldopa	amino acids, peptides, and analogues	C10H13NO4	211.2145	6.04	neg
4-guanidinobutanoic acid	amino acids, peptides, and analogues	C5H11N3O2	145.1597	1.66	pos
5-methylcytosine	pyrimidines and pyrimidine derivatives	C5H7N3O	125.1286	1.21	pos
acetylglycine	amino acids, peptides, and analogues	C4H7NO3	117.1033	1.31	neg
adenine	purines and purine derivatives	C5H5N5	135.1267	1.61	pos, neg
adenosine 5'-monophosphate	nucleosides, nucleotides, and analogues	C10H14N5O7P	347.2212	1.77	pos, neg
alanine / sarcosine	amino acids, peptides, and analogues	C3H7NO2	89.0932	0.75	pos
allantoin	imidazoles	C4H6N4O3	158.1154	0.85	neg
arginine	amino acids, peptides, and analogues	C6H14N4O2	174.2010	0.74	pos
aspartic acid	amino acids, peptides, and analogues	C4H7NO4	133.1027	0.74	pos
betaine	amino acids, peptides, and analogues	C5H11NO2	117.1463	0.86	pos
carnitine	quaternary ammonium salts	C7H15NO3	161.1989	1.01	pos
citramalic acid	fatty acids and conjugates	C5H8O5	148.1140	2.38	neg

citric acid	organic acids and derivatives	C6H8O7	192.1235	2.05	pos, neg
cytidine	nucleosides, nucleotides, and analogues	C9H13N3O5	243.2166	1.69	pos, neg
cytosine	pyrimidines and pyrimidine derivatives	C4H5N3O	111.1020	0.89	pos
dihydrouracil	pyrimidines and pyrimidine derivatives	C4H6N2O2	114.1026	1.43	pos
dopamine	catecholamines and derivatives	C8H11NO2	153.1784	2.86	pos, neg
fumaric acid	organic acids and derivatives	C4H4O4	116.0722	2.08	neg
gamma-aminobutyric acid	amino acids, peptides, and analogues	C4H9NO2	103.1198	0.83	pos
gluconic acid	carbohydrates and carbohydrate conjugates	C6H12O7	196.1553	0.78	pos, neg
glucosamine	carbohydrates and carbohydrate conjugates	C6H13NO5	179.1711	0.71	pos
glucose / fructose	carbohydrates and carbohydrate conjugates	C6H12O6	180.1559	0.80	neg
glucuronic acid / galacturonic acid	carbohydrates and carbohydrate conjugates	C6H10O7	194.1394	0.78	neg
glutamic acid	amino acids, peptides, and analogues	C5H9NO4	147.1293	0.76	neg
glutamine	amino acids, peptides, and analogues	C5H10N2O3	146.1445	0.76	pos, neg
glyceraldehyde	carbohydrates and carbohydrate conjugates	C3H6O3	90.0779	0.80	neg
glyceric acid	carbohydrates and carbohydrate conjugates	C3H6O4	106.0773	0.91	neg
glycine	amino acids, peptides, and analogues	C2H5NO2	75.0666	0.74	pos
glycolic acid	organic acids and derivatives	C2H4O3	76.0514	0.93	neg
guanosine	nucleosides, nucleotides, and analogues	C10H13N5O5	283.2407	6.40	neg

hippuric acid	benzene and substituted derivatives	C9H9NO3	179.1727	7.93	pos, neg
histamine	amines	C5H9N3	111.1451	0.66	pos
homovanillic acid	phenols	C9H10O4	182.1733	7.65	neg
hypoxanthine	purines and purine derivatives	C5H4N4O	136.1115	2.15	pos, neg
imidazoleacetic acid	imidazoles	C5H6N2O2	126.1133	1.01	pos
isocitric acid	organic acids and derivatives	C6H8O7	192.1235	1.21	pos, neg
isoleucine	amino acids, peptides, and analogues	C6H13NO2	131.1729	2.40	pos
kynurenic acid	quinolines and derivatives	C10H7NO3	189.1675	7.66	pos
lactic acid	organic acids and derivatives	C3H6O3	90.0779	0.84	pos
L-Dopa (levodopa)	amino acids, peptides, and analogues	C9H11NO4	197.1879	2.36	pos
leucine	amino acids, peptides, and analogues	C6H13NO2	131.1729	2.63	pos
malic acid	organic acids and derivatives	C4H6O5	134.0874	1.14	neg
malonic acid	organic acids and derivatives	C3H4O4	104.0615	1.30	neg
methionine	amino acids, peptides, and analogues	C5H11NO2S	149.2110	1.51	pos
N-acetylaspartic acid	amino acids, peptides, and analogues	C6H9NO5	175.1394	1.58	pos, neg
N-acetylglutamic acid	amino acids, peptides, and analogues	C7H11NO5	189.1659	2.32	pos, neg
N-acetylputrescine	organic acids and derivatives	C6H14N2O	130.1882	1.18	pos
N-alpha-acetylarginine	amino acids, peptides, and analogues	C8H16N4O3	216.2376	1.60	pos
N-alpha-acetyllysine	amino acids, peptides, and analogues	C8H16N2O3	188.22424	1.11	pos
nicotinic acid	pyridines and derivatives	C6H5NO2	123.1094	1.52	neg
N-methyl aspartic acid	amino acids, peptides, and analogues	C5H9NO4	147.1293	0.80	pos
norepinephrine	catecholamines and derivatives	C8H11NO3	169.1778	1.03	pos, neg

ornithine	amino acids, peptides, and analogues	C5H12N2O2	132.1610	0.66	pos
pantothenic acid	alcohols and polyols	C9H17NO5	219.2350	6.60	pos
phenylalanine	amino acids, peptides, and analogues	C9H11NO2	165.1891	6.20	pos
phenylethylamine	amines	C8H11N	121.1796	6.71	pos
pipecolic acid	amino acids, peptides, and analogues	C6H11NO2	129.1570	1.30	pos
proline	amino acids, peptides, and analogues	C5H9NO2	115.1305	0.91	pos, neg
pyridoxal	pyridines and derivatives	C8H9NO3	167.1620	1.98	pos
pyroglutamic acid	amino acids, peptides, and analogues	C5H7NO3	129.1140	1.94	pos, neg
quinic acid	alcohols and polyols	C7H12O6	192.1666	0.90	neg
serine	amino acids, peptides, and analogues	C3H7NO3	105.0926	0.72	pos
serotonin	tryptamines and derivatives	C10H12N2O	176.2151	6.31	pos
succinic acid	organic acids and derivatives	C4H6O4	118.0880	2.39	pos, neg
tartaric acid	carbohydrates and carbohydrate conjugates	C4H6O6	150.0868	0.92	neg
taurine	amino acids, peptides, and analogues	C2H7NO3S	125.1470	0.75	pos
thymine	pyrimidines and pyrimidine derivatives	C5H6N2O2	126.1133	3.395	pos
tryptophan	amino acids, peptides, and analogues	C11H12N2O2	204.2252	7.60	pos, neg
tryptophanamide	tryptamines and derivatives	C11H13N3O	203.2404	6.81	pos
tyramine	amines	C8H11NO	137.1790	4.45	pos
tyrosine	amino acids, peptides, and analogues	C9H11NO3	181.1885	3.43	pos, neg
uracil	pyrimidines and pyrimidine derivatives	C4H4N2O2	112.0868	1.49	pos, neg
ureidopropionic acid	amino acids, peptides, and analogues	C4H8N2O3	132.1179	1.30	pos
uric acid	purines and purine derivatives	C5H4N4O3	168.1103	1.92	pos, neg

uridine	nucleosides, nucleotides, and analogues	C9H12N2O6	244.2014	2.90	pos, neg
uridine 5'-monophosphate	nucleosides, nucleotides, and analogues	C9H13N2O9P	324.1813	1.37	pos, neg
valine	amino acids, peptides, and analogues	C5H11NO2	117.1463	1.18	pos, neg
vanilloloside	carbohydrates and carbohydrate conjugates	C14H20O8	316.3060	7.89	neg
xanthine	purines and purine derivatives	C5H4N4O2	152.1109	2.60	neg

Supplementary Table S2. A list of metabolites determined using AbsoluteIDQ p180 kit in different honeybee venom extracts (methanolic; 0.1% formic acid in water; DMSO). "<LOD" means a value below limit of detection; "<LLOQ" represents a value below lower limit of quantitation; ">ULOQ" means a value above upper limit of quantitation.

Class	Metabolite	% dry weight of honeybee venom		
		methanol	0.1% formic acid	DMSO
acylcarnitines	carnitine	2.73E-04	2.77E-04	4.27E-04
	acetylcarnitine	<LLOQ	<LLOQ	<LLOQ
	propionylcarnitine	<LOD	<LOD	<LOD
	hydroxybutyrylcarnitine	<LOD	<LOD	<LOD
	hydroxypropionylcarnitine	<LOD	<LOD	<LOD
	propenoylcarnitine	1.23E-06	<LOD	<LOD
	butyrylcarnitine	<LOD	<LOD	<LOD
	butenylcarnitine	2.22E-06	<LOD	<LOD
	valerylcarnitine	<LOD	<LOD	<LOD
	glutarylcarnitine(hydroxyhexanoylcarnitine)	<LOD	<LOD	<LOD
	methylglutarylcarnitine	<LOD	<LOD	<LOD
	hydroxyvalerylcarnitine (methylmalonylcarnitine)	<LOD	<LOD	<LOD
	tiglylcarnitine	<LOD	<LOD	<LOD
	glutaconylcarnitine	<LOD	<LOD	6.74E-06
	hexanoylcarnitine (fumarylcarnitine)	<LOD	<LOD	<LOD
	hexenoylcarnitine	9.06E-06	4.75E-06	2.59E-05
	pimelylcarnitine	<LOD	<LOD	<LOD
	octanoylcarnitine	<LOD	<LOD	<LOD
	nonaylcarnitine	<LOD	<LOD	<LOD
	decanoylcarnitine	<LLOQ	<LLOQ	<LLOQ
	decenoylcarnitine	<LOD	<LOD	<LOD
	decadienylcarnitine	<LOD	<LOD	<LOD
	dodecanoylcarnitine	<LLOQ	<LLOQ	<LLOQ
	dodecanedioylcarnitine	5.23E-05	<LOD	<LOD
	dodecenoylcarnitine	1.34E-05	7.77E-06	1.56E-05
	tetradecanoylcarnitine	<LLOQ	<LLOQ	<LLOQ
	tetradecenoylcarnitine	2.51E-06	1.11E-06	2.27E-06
	hydroxytetradecenoylcarnitine	1.85E-06	<LOD	3.16E-06
	tetradecadienylcarnitine	1.32E-06	<LOD	<LOD
	hydroxytetradecadienylcarnitine	1.90E-06	<LOD	<LOD
	hexadecanoylcarnitine	<LLOQ	<LLOQ	<LLOQ
	hydroxyhexadecanoylcarnitine	4.03E-05	1.15E-05	2.55E-05
	hexadecenoylcarnitine	<LOD	<LOD	<LOD
	hydroxyhexadecenoylcarnitine	3.53E-06	2.06E-06	3.34E-06
	hexadecadienylcarnitine	2.06E-06	<LOD	2.31E-06
	hydroxyhexadecadienylcarnitine	<LOD	<LOD	<LOD
	octadecanoylcarnitine	<LLOQ	<LLOQ	<LLOQ

	octadecenoylcarnitine	<LOD	<LOD	<LOD
	hydroxyoctadecenoylcarnitine	4.67E-06	<LOD	4.33E-06
	octadecadienylcarnitine	2.55E-06	1.42E-06	2.81E-06
amino acids and biogenic amines	alanine	8.65E-03	1.44E-02	9.25E-03
	arginine	1.44E-03	>ULOQ	>ULOQ
	asparagine	<LLOQ	2.76E-04	3.38E-04
	aspartate	3.09E-04	4.24E-03	2.03E-03
	citrulline	<LLOQ	<LLOQ	<LLOQ
	glutamine	<LOD	<LOD	<LLOQ
	glutamate	1.11E-03	2.75E-03	1.35E-03
	glycine	1.39E-02	1.45E-02	1.08E-02
	histidine	3.12E-04	1.36E-03	1.17E-03
	isoleucine	8.17E-04	9.65E-04	7.94E-04
	leucine	<LLOQ	<LLOQ	<LLOQ
	lysine	3.33E-03	5.28E-03	3.91E-03
	methionine	<LOD	<LOD	<LLOQ
	ornithine	<LLOQ	<LLOQ	1.83E-04
	phenylalanine	3.91E-04	5.94E-04	5.47E-04
	proline	>ULOQ	>ULOQ	>ULOQ
	serine	2.97E-04	7.18E-04	7.01E-04
	threonine	2.45E-04	5.30E-04	4.13E-04
	tryptophan	<LLOQ	3.68E-04	3.46E-04
	tyrosine	2.69E-04	4.71E-04	4.16E-04
	valine	9.27E-04	1.01E-03	9.24E-04
	acetylornithine	4.01E-04	4.64E-04	1.27E-04
	asymmetric dimethylarginine	1.12E-04	1.97E-04	1.41E-04
	alpha-aminoadipic acid	<LOD	<LOD	<LOD
	cis-4-hydroxyproline	<LOD	<LLOQ	<LOD
	carnosine	<LOD	<LLOQ	<LLOQ
	creatinine	<LLOQ	<LLOQ	2.42E-04
	L-Dopa	<LOD	2.13E-05	<LOD
	dopamine	>ULOQ	>ULOQ	>ULOQ
	histamine	>ULOQ	>ULOQ	>ULOQ
	kynurenine	4.00E-05	4.75E-05	6.85E-05
	methioninesulfoxide	<LLOQ	<LLOQ	<LLOQ
	nitrotyrosine	<LOD	<LOD	<LOD
	phenylethylamine	1.76E-05	3.36E-05	1.71E-05
	putrescine	>ULOQ	>ULOQ	>ULOQ
	symmetric dimethylarginine	<LLOQ	<LOD	<LLOQ
	serotonin	>ULOQ	>ULOQ	>ULOQ
	spermidine	2.84E-04	>ULOQ	>ULOQ
	spermine	<LLOQ	8.15E-05	1.59E-05
	trans-4-hydroxyproline	<LLOQ	<LLOQ	3.59E-04
	taurine	2.24E-03	>ULOQ	>ULOQ
	total dimethylarginine	6.85E-05	1.23E-04	8.84E-05

hexoses	hexoses including glucose	2.14E-01	4.37E-01	4.87E-01
glycerophospholipids [a]	lysoPC a C14:0	<LOD	<LOD	<LOD
	lysoPC a C16:0	6.81E-05	6.05E-05	1.45E-04
	lysoPC a C16:1	<LOD	<LOD	1.12E-05
	lysoPC a C17:0	9.73E-06	8.26E-06	1.61E-05
	lysoPC a C18:0	1.03E-04	6.19E-05	1.21E-04
	lysoPC a C18:1	1.26E-04	1.08E-04	1.51E-04
	lysoPC a C18:2	7.79E-05	5.57E-05	1.09E-04
	lysoPC a C20:3	<LOD	<LOD	2.57E-05
	lysoPC a C20:4	5.02E-06	4.84E-06	1.57E-05
	lysoPC a C24:0	<LOD	<LOD	<LOD
	lysoPC a C26:0	<LOD	<LOD	9.53E-06
	lysoPC a C26:1	<LOD	<LOD	1.04E-05
	lysoPC a C28:0	<LOD	<LOD	<LOD
	lysoPC a C28:1	<LOD	<LOD	9.27E-06
	PC aa C24:0	<LOD	<LOD	<LOD
	PC aa C26:0	<LOD	<LOD	<LOD
	PC aa C28:1	<LOD	<LOD	<LOD
	PC aa C30:0	3.74E-05	3.06E-05	4.17E-05
	PC aa C30:2	1.92E-06	1.67E-06	4.33E-06
	PC aa C32:0	1.35E-05	4.13E-06	3.38E-05
	PC aa C32:1	1.83E-05	4.28E-06	3.88E-05
	PC aa C32:2	1.93E-06	1.56E-06	5.95E-06
	PC aa C32:3	2.16E-06	9.45E-07	5.23E-06
	PC aa C34:1	4.73E-05	<LOD	4.41E-04
	PC aa C34:2	<LOD	<LOD	7.24E-04
	PC aa C34:3	2.44E-05	<LOD	4.97E-05
	PC aa C34:4	4.21E-06	1.57E-06	1.14E-05
	PC aa C36:0	<LOD	<LOD	<LOD
	PC aa C36:1	2.35E-05	<LOD	1.28E-04
	PC aa C36:2	3.65E-05	<LOD	5.21E-04
	PC aa C36:3	2.69E-05	<LOD	2.27E-04
	PC aa C36:4	2.04E-05	<LOD	3.50E-04
	PC aa C36:5	8.95E-06	<LOD	3.83E-05
	PC aa C36:6	1.48E-05	<LOD	1.93E-05
	PC aa C38:0	<LOD	<LOD	1.23E-05
	PC aa C38:1	2.03E-06	<LOD	4.79E-06
	PC aa C38:3	<LOD	<LOD	1.05E-04
	PC aa C38:4	<LOD	<LOD	2.25E-04
	PC aa C38:5	<LOD	<LOD	8.36E-05
	PC aa C38:6	4.43E-06	<LOD	1.66E-04
	PC aa C40:1	<LOD	<LOD	<LOD
	PC aa C40:2	3.51E-06	<LOD	1.06E-05
	PC aa C40:3	2.99E-06	<LOD	5.57E-06
	PC aa C40:4	1.85E-06	<LOD	7.44E-06

	PC aa C40:5	<LOD	<LOD	1.96E-05
	PC aa C40:6	<LOD	<LOD	6.56E-05
	PC aa C42:0	<LOD	<LOD	<LOD
	PC aa C42:1	<LOD	<LOD	8.98E-06
	PC aa C42:2	1.94E-05	<LOD	2.91E-05
	PC aa C42:4	2.35E-06	<LOD	3.89E-06
	PC aa C42:5	<LOD	<LOD	<LOD
	PC aa C42:6	<LOD	<LOD	<LOD
	PC ae C30:0	<LOD	<LOD	<LOD
	PC ae C30:1	1.46E-06	<LOD	3.12E-06
	PC ae C30:2	<LOD	<LOD	<LOD
	PC ae C32:1	2.01E-06	<LOD	7.32E-06
	PC ae C32:2	<LOD	<LOD	<LOD
	PC ae C34:0	1.61E-05	5.31E-06	2.02E-05
	PC ae C34:1	9.58E-06	3.47E-06	2.69E-05
	PC ae C34:2	2.99E-06	<LOD	3.29E-05
	PC ae C34:3	5.55E-06	<LOD	2.11E-05
	PC ae C36:0	<LOD	<LOD	<LOD
	PC ae C36:1	<LOD	<LOD	2.87E-05
	PC ae C36:2	<LOD	<LOD	4.71E-05
	PC ae C36:3	4.53E-06	<LOD	2.07E-05
	PC ae C36:4	<LOD	<LOD	3.88E-05
	PC ae C36:5	2.36E-06	<LOD	2.35E-05
	PC ae C38:0	<LOD	<LOD	<LOD
	PC ae C38:1	<LOD	<LOD	<LOD
	PC ae C38:2	1.07E-05	2.26E-06	6.01E-06
	PC ae C38:3	4.20E-06	<LOD	1.24E-05
	PC ae C38:4	<LOD	<LOD	3.07E-05
	PC ae C38:5	<LOD	<LOD	3.26E-05
	PC ae C38:6	1.78E-06	<LOD	1.91E-05
	PC ae C40:1	2.59E-06	<LOD	6.45E-06
	PC ae C40:2	3.67E-06	1.22E-06	1.01E-05
	PC ae C40:3	<LOD	<LOD	3.81E-06
	PC ae C40:4	<LOD	<LOD	7.09E-06
	PC ae C40:5	<LOD	<LOD	7.89E-06
	PC ae C40:6	1.15E-05	2.40E-06	3.39E-05
	PC ae C42:0	<LOD	<LOD	<LOD
	PC ae C42:1	<LOD	<LOD	<LOD
	PC ae C42:2	2.63E-06	<LOD	1.06E-05
	PC ae C42:3	1.29E-06	<LOD	4.19E-06
	PC ae C42:4	<LOD	<LOD	<LOD
	PC ae C42:5	<LOD	<LOD	<LOD
	PC ae C44:3	<LOD	<LOD	6.25E-06
	PC ae C44:4	<LOD	<LOD	<LOD
	PC ae C44:5	<LOD	<LOD	<LOD
	PC ae C44:6	<LOD	<LOD	6.71E-06

sphingolipids [b]	SM (OH) C14:1	<LOD	<LOD	1.21E-05
	SM (OH) C16:1	1.94E-05	8.93E-06	2.24E-05
	SM (OH) C22:1	<LOD	<LOD	2.77E-05
	SM (OH) C22:2	<LOD	<LOD	3.03E-05
	SM (OH) C24:1	<LOD	<LOD	5.49E-06
	SM C16:0	3.13E-04	1.63E-04	4.65E-04
	SM C16:1	7.03E-06	3.46E-06	3.17E-05
	SM C18:0	1.05E-03	4.44E-04	9.22E-04
	SM C18:1	4.52E-05	2.43E-05	5.93E-05
	SM C20:2	<LOD	<LOD	8.19E-07
	SM C22:3	8.02E-07	<LOD	4.39E-06
	SM C24:0	2.63E-06	<LOD	4.01E-05
	SM C24:1	<LOD	<LOD	1.17E-04
	SM C26:0	<LOD	<LOD	1.51E-06
	SM C26:1	<LOD	<LOD	1.53E-06

[a] PC aa/ae C *x*:*y* – phosphatidylcholine; where ‘aa’ denotes diacyl phosphatidylcholine, in turn ‘ae’ acyl-alkyl phosphatidylcholine, both with sum of carbon atoms and double bonds present in both lipid fatty acid chains indicated as *x* and *y*, respectively

[b] SM (OH) C *x*:*y* – sphingomyelin (with hydroxyl group denotes ‘OH’) with the sum of carbon atoms of both chains indicated as *x* and the total number of double bonds described as *y*

Supplementary Table S3. A list of MRM transitions for 42 amino acids and their corresponding internal standards along with collision energy values. Collision cell potential (CXP) of 50 V was applied for each transition.

AMINO ACID	ABBREV.	MRM TRANSITION (Q1 > Q3)				COLLISION ENERGY (EV)
		ANALYTE		INTERNAL STD		
		Q1	Q3	Q1	Q3	
1-methylhistidine	1MHis	318.2	121.1	310.2	113.1	30
3-methylhistidine	3MHis	318.2	121.1	310.2	113.1	30
alanine	Ala	238.2	121.1	230.2	113.1	30
anserine	Ans	389.2	121.1	381.2	113.1	30
arginine	Arg	323.2	121.1	315.2	113.1	30
argininosuccinic acid	Asa	439.2	121.1	431.2	113.1	50
asparagine	Asn	281.2	121.1	273.2	113.1	30
aspartic acid	Asp	282.1	121.1	274.1	113.1	30
carnosine	Car	375.2	121.1	367.2	113.1	30
citrulline	Cit	324.2	121.1	316.2	113.1	30
cystathionine	Cth	519.3	121.1	503.3	113.1	50
cystine	Cys	537.2	121.1	521.2	113.1	50
ethanolamine	EtN	210.2	121.1	202.2	113.1	30
glutamic acid	Glu	296.2	121.1	288.2	113.1	30
glutamine	Gln	295.2	121.1	287.2	113.1	30
glycine	Gly	224.1	121.1	216.1	113.1	30
histidine	His	304.2	121.1	296.2	113.1	30
homocitrulline	Hcit	338.2	121.1	330.2	113.1	30
homocystine	Hcy	565.3	121.1	549.3	113.1	50
hydroxyproline	Hyp	280.1	121.1	272.1	113.1	30
isoleucine	Ile	280.2	121.1	272.2	113.1	30
leucine	Leu	280.2	121.1	272.2	113.1	30
lysine	Lys	443.3	121.1	427.3	113.1	50
methionine	Met	298.2	121.1	290.2	113.1	30
ornithine	Orn	429.3	121.1	413.3	113.1	50
phenylalanine	Phe	314.2	121.1	306.2	113.1	30
phosphoethanolamine	PEtN	290.1	121.1	282.1	113.1	30
phosphoserine	PSer	334.1	121.1	326.1	113.1	30
proline	Pro	264.2	121.1	256.2	113.1	30
sarcosine	Sar	238.2	121.1	230.2	113.1	30
serine	Ser	254.2	121.1	246.2	113.1	30

taurine	Tau	274.1	121.1	266.1	113.1	30
threonine	Thr	268.2	121.1	260.2	113.1	30
tryptophan	Trp	353.2	121.1	345.2	113.1	30
tyrosine	Tyr	330.2	121.1	322.2	113.1	30
valine	Val	266.2	121.1	258.2	113.1	30
α-aminoadipic acid	Aad	310.2	121.1	302.2	113.1	30
α-amino-n-butyric acid	Abu	252.2	121.1	244.2	113.1	30
β-alanine	bAla	238.2	121.1	230.2	113.1	30
β-aminoisobutyric acid	bAib	252.2	121.1	244.2	113.1	30
γ-amino-n-butyric acid	GABA	252.2	121.1	244.2	113.1	30
δ-hydroxylysine	Hyl	459.3	121.1	443.3	113.1	50

Supplementary Table S4. The percentage content of the amino acids quantified in the studied honeybee venom samples collected in different years (percentage of the dry weight of the venom). Venom samples were stored at -80°C until analysis in 2019.

PROTEINOGENIC AMINO ACIDS (% dry weight of honeybee venom)				
Name	Year of sample collection			
	2019	2018	2017	2015
alanine	0.0201	0.0300	0.0236	0.0721
arginine	0.0173	0.0210	0.0239	0.0395
asparagine	0.0071	0.0063	0.0036	0.0196
aspartatic acid	0.0043	0.0061	0.0071	0.0232
glutamic acid	0.0264	0.0273	0.0210	0.1163
glutamine	0.0219	0.0161	0.0171	0.0443
glycine	0.0090	0.0124	0.0102	0.0182
histidine	0.0042	0.0033	0.0046	0.0261
isoleucine	0.0040	0.0071	0.0058	0.0131
leucine	0.0047	0.0078	0.0061	0.0184
lysine	0.0090	0.0159	0.0126	0.0241
methionine	0.0049	0.0057	0.0041	0.0053
phenylalanine	0.0038	0.0054	0.0042	0.0130
proline	0.0526	0.1050	0.0994	0.3152
serine	0.0063	0.0084	0.0051	0.0231
threonine	0.0045	0.0062	0.0048	0.0129
tryptophan	0.0024	0.0025	0.0016	0.0045
tyrosine	0.0027	0.0051	0.0047	0.0086
valine	0.0072	0.0112	0.0066	0.0196
total	0.2123	0.3028	0.2660	0.8171
NON-PROTEINOGENIC AMINO ACIDS (% dry weight of honeybee venom)				
Name	Year of sample collection			
	2019	2018	2017	2015
α-aminoadipic acid	0.0004	0.0002	0.0002	0.0009
α-amino-n-butyric	0.0007	0.0025	0.0045	0.0046

β-aminoisobutyric	0.0018	0.0018	0.0023	0.0012
β-alanine	0.0247	0.0287	0.0270	0.0493
cystine	0.0232	0.0128	0.0212	0.0075
ethanolamine	0.0152	0.0130	0.0116	0.0098
γ-amino-n-butyric acid (GABA)	0.0040	0.0044	0.0033	0.0096
hydroxyproline	0.0008	0.0011	0.0023	0.0059
ornithine	0.0012	0.0015	0.0014	0.0017
phosphoethanolamine	0.0052	0.0124	0.0083	0.0094
sarcosine	0.0009	0.0018	0.0017	0.0057
taurine	0.0392	0.0289	0.0231	0.0473
total	0.1173	0.1091	0.1069	0.1529

Supplementary Table S5. LC parameters for untargeted metabolomics.

<table>
<tr><th>PARAMETER</th><th>SETTING</th></tr>
<tr><td>Chromatographic column</td><td>ACE Excel 2 C18-PFP, 2.1×100 mm, 2,0 µm
(Advanced Chromatography Technologies Ltd)</td></tr>
<tr><td>Mobile phase</td><td>Eluent A: 0.1% formic acid in water
Eluent B: acetonitrile</td></tr>
<tr><td>Flow rate</td><td>0.35 mL/min</td></tr>
<tr><td>Temperature</td><td>25 °C</td></tr>
<tr><td>Gradient elution profile</td><td><table>
<tr><td>Total time, min</td><td>% A</td></tr>
<tr><td>0</td><td>100</td></tr>
<tr><td>3</td><td>100</td></tr>
<tr><td>13</td><td>20</td></tr>
<tr><td>16</td><td>20</td></tr>
<tr><td>16.5</td><td>100</td></tr>
<tr><td>20.5</td><td>100</td></tr>
</table></td></tr>
<tr><td>Injection volume</td><td>2 µL for positive ionization
4 µL for negative ionization</td></tr>
<tr><td>Run time</td><td>20.5 min</td></tr>
<tr><td>Detector</td><td>Q-Orbirap mass spectrometer</td></tr>
</table>

Supplementary Table S6. MS parameters for untargeted metabolomics.

PARAMETER	SETTING
Ionization method	Electrospray
Ionization mode	Positive and negative
Scan mode	Data dependent MS/MS (top 5) with full MS
Mass range	70-1,000 m/z
Probe temperature	350 °C
Spray voltage	3,500 V/-3,500 V
Capillary temperature	320 °C
Sheath gas flow	40 arb
Auxiliary gas flow	10 arb
Sweep gas flow	1 arb
Mass resolution	35,000 at *m/z* 200
Source collision-induced dissociation	2 eV

Supplementary Table S7. LC parameters for wide-spectrum metabolite determination using AbsoluteIDQ p180 kit.

<table>
<tr><th colspan="2">LC-MS/MS
(Determination of amino acids and biogenic amines)</th></tr>
<tr><th>PARAMETER</th><th>SETTING</th></tr>
<tr><td>Chromatographic column</td><td>Zorbax Eclipse XDB C18, 3.0×100 mm, 3.5 µm (Agilent Technologies)</td></tr>
<tr><td>Pre-column</td><td>SecurityGuard C18 (3.0×4.0 mm, Phenomenex)</td></tr>
<tr><td>Mobile phase</td><td>Eluent A: 0.2% formic acid in water
Eluent B: 0.2% formic acid in acetonitrile</td></tr>
<tr><td>Flow rate</td><td>0.5 mL/min</td></tr>
<tr><td>Temperature</td><td>50 °C</td></tr>
<tr><td>Gradient elution profile</td><td><table><tr><th>Total time, min</th><th>% A</th></tr><tr><td>0</td><td>100</td></tr><tr><td>0.5</td><td>100</td></tr><tr><td>5.5</td><td>5</td></tr><tr><td>6.5</td><td>5</td></tr><tr><td>7</td><td>100</td></tr><tr><td>9.5</td><td>100</td></tr></table></td></tr>
<tr><td>Injection volume</td><td>10 µL</td></tr>
<tr><td>Run time</td><td>9.5 min</td></tr>
<tr><td>Detector</td><td>Triple quadrupole mass spectrometer</td></tr>
<tr><th colspan="2">FIA-MS/MS
(Determination of hexoses, acylcarnitines, sphingomyelins and glycerophospholipid)s</th></tr>
<tr><th>PARAMETER</th><th>SETTING</th></tr>
<tr><td>Chromatographic column</td><td>None. Flow injection analysis (FIA)</td></tr>
<tr><td>Mobile phase</td><td>Biocrates MS Running Solvent</td></tr>
<tr><td>Flow rate</td><td>0.03 – 0.2 mL/min</td></tr>
<tr><td>Injection volume</td><td>20 µL</td></tr>
<tr><td>Run time</td><td>3 min</td></tr>
<tr><td>Detector</td><td>Triple quadrupole mass spectrometer</td></tr>
</table>

Supplementary Table S8. MS parameters for wide-spectrum metabolite determination using AbsoluteIDQ p180 kit.

PARAMETER	SETTING	
	LC-MS/MS	FIA-MS/MS
Ionization method	Electrospray	
Ionization mode	Positive	Positive and negative
Scan mode	Multiple reaction monitoring (MRM)	
Temperature	500 °C	200 °C
Ion spray voltage	5500 V	5500 V for positive ionization *<br>-4500 V for negative ionization **
Curtain gas	20 psig	
Ion source gas 1	40 psig	
Ion source gas 2	50 psig	
Entrance potential	10 V	10 V / -10 V
Collison gas	Nitrogen	

*positive ionization mode was used for determination of amino acids, amines, acylocarnitines, glycerophospholipids, and sphingolipids
**negative ionization was used for determination of hexoses

Supplementary Table S9. LC parameters for wide-spectrum amino acid determination using aTRAQ kit.

<table>
<tr><th>PARAMETER</th><th>SETTING</th></tr>
<tr><td>Chromatographic column</td><td>AAA C18, 4.6×150 mm (Sciex)</td></tr>
<tr><td>Mobile phase</td><td>Eluent A: 0.1% formic acid and 0.01% heptafluorobutyric acid in water
Eluent B: 0.1% formic acid and 0.01% heptafluorobutyric acid in methanol</td></tr>
<tr><td>Flow rate</td><td>0.8 mL/min</td></tr>
<tr><td>Temperature</td><td>50 °C</td></tr>
<tr><td>Gradient elution profile</td><td><table>
<tr><th>Total time, min</th><th>% A</th></tr>
<tr><td>0</td><td>98</td></tr>
<tr><td>6</td><td>60</td></tr>
<tr><td>10</td><td>60</td></tr>
<tr><td>11</td><td>10</td></tr>
<tr><td>12</td><td>10</td></tr>
<tr><td>13</td><td>98</td></tr>
<tr><td>18</td><td>98</td></tr>
</table></td></tr>
<tr><td>Injection volume</td><td>2 µL</td></tr>
<tr><td>Run time</td><td>18 min</td></tr>
<tr><td>Detector</td><td>Triple quadrupole mass spectrometer</td></tr>
</table>

Supplementary Table S10. MS parameters for wide-spectrum amino acid determination using aTRAQ kit.

PARAMETER	SETTING
Ionization method	Electrospray
Ionization mode	Positive
Scan mode	Multiple reaction monitoring (MRM)
Temperature	600 °C
Ion spray voltage	4500 V
Curtain gas	20 psig
Ion source gas 1	60 psig
Ion source gas 2	50 psig
Declustering potential	30 V
Entrance potential	10 V
Collison gas	Nitrogen

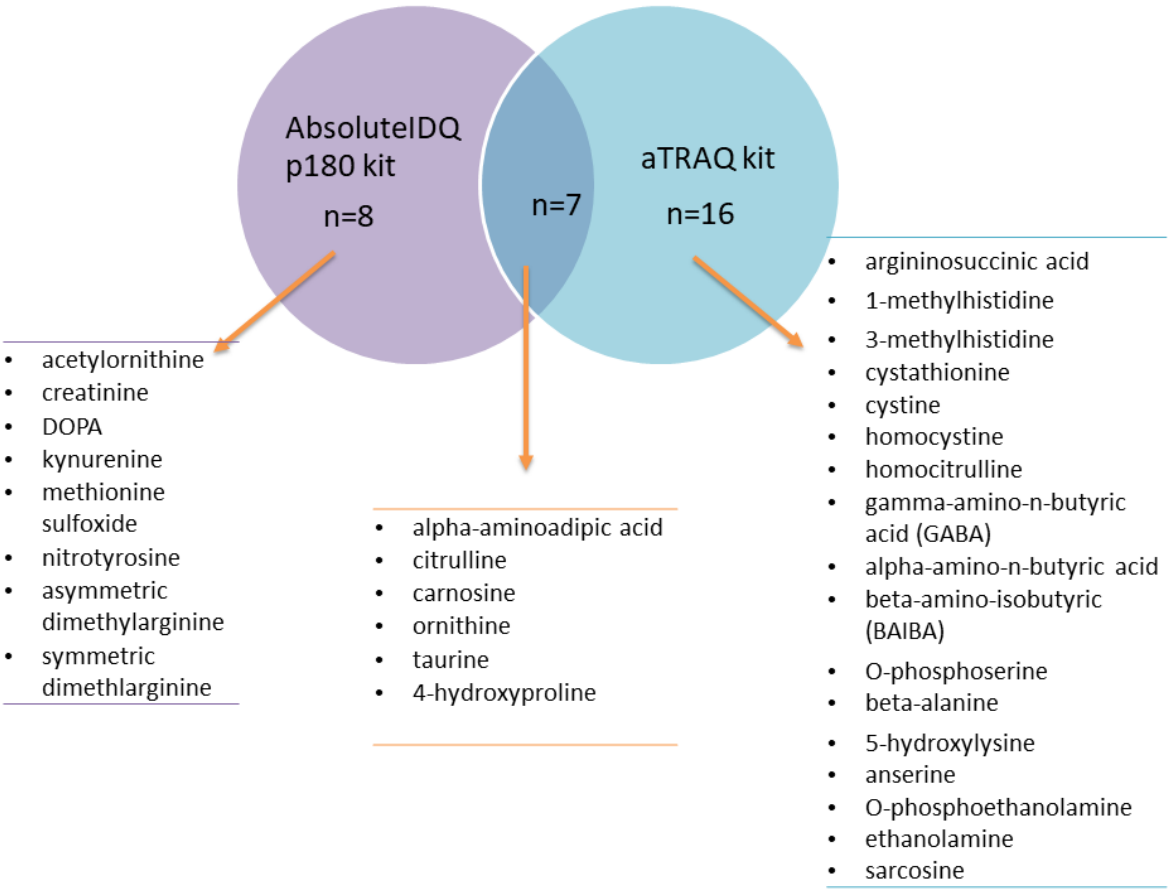

Supplementary Figure S1. Venn diagram presenting common and unique analytes covered by the two applied targeted metabolomics methodologies (AbsoluteIDQ p180 kit and aTRAQ kit) among non-proteinogenic amino acids and amino acid derivatives. Both kits enable an analysis of the same set of proteinogenic amino acids.

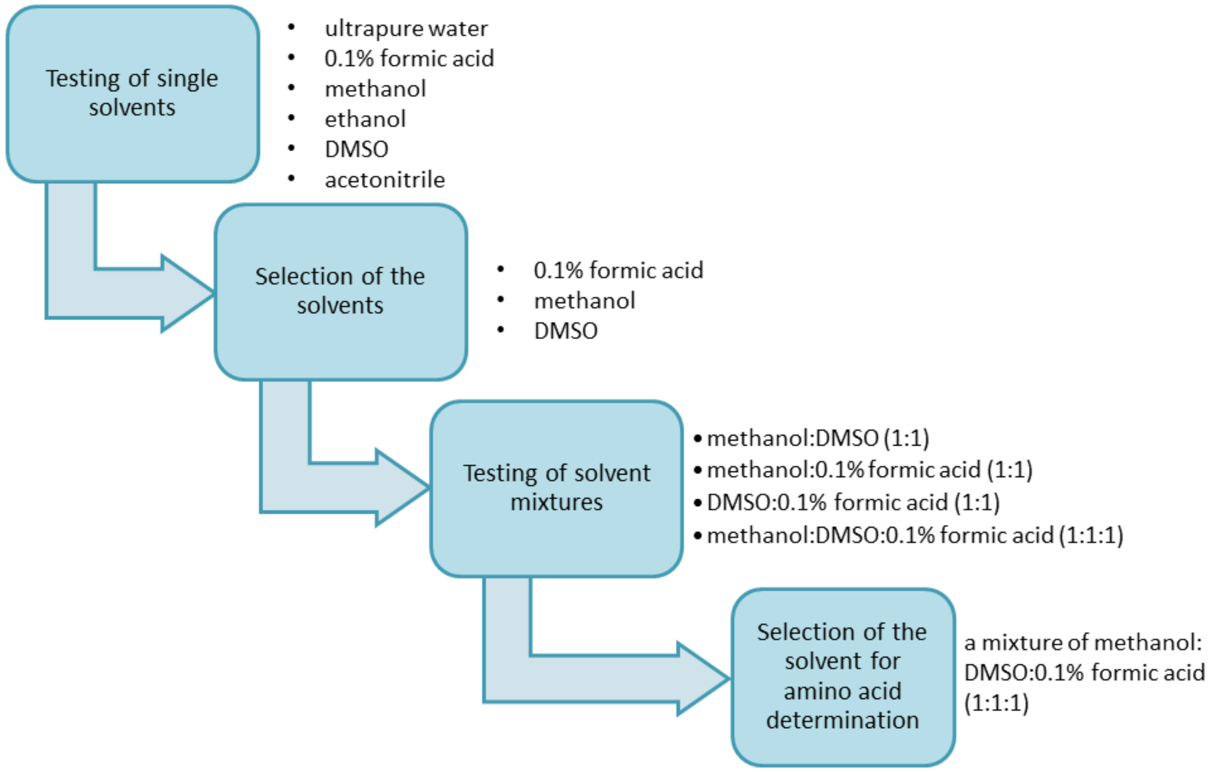

Supplementary Figure S2. A workflow applied to select the most appropriate solvent for the determination of free amino acids in honeybee venom samples. Two criteria for choosing a solvent were set up: number of analytes quantified in a sample (with concentrations above the lower limit of quantitation) and a total amino acid concentration.

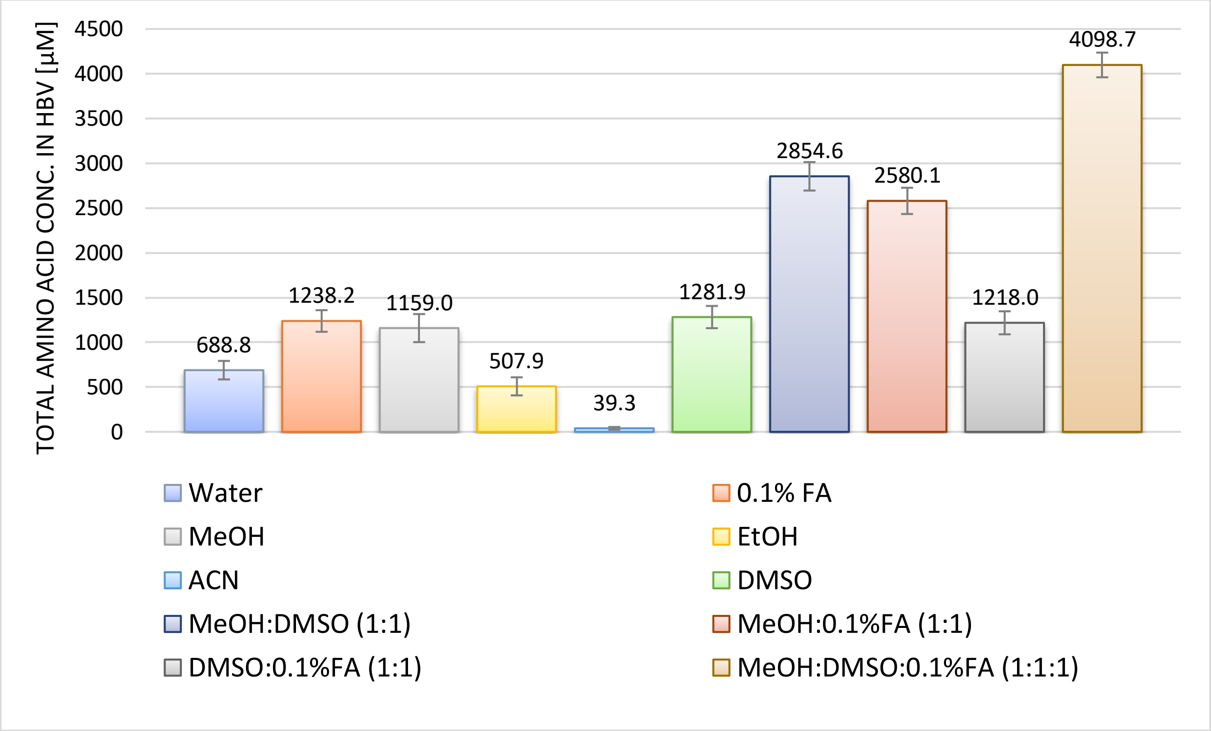

Supplementary Figure S3. Bar chart showing the impact of solvent on the total amino acid concentration (average ± SD) quantified in honeybee venom sample. Among single solvents, the highest total amino acid concentration was observed using DMSO, 0.1% formic acid in water, and methanol. Therefore, the mixtures of these three solvents were tested in the next step of method optimization. MeOH – methanol, ACN – acetonitrile, EtOH – ethanol, DMSO – dimethyl sulfoxide, 0.1%FA – 0.1% formic acid in water. In brackets volume ratios were given.

A

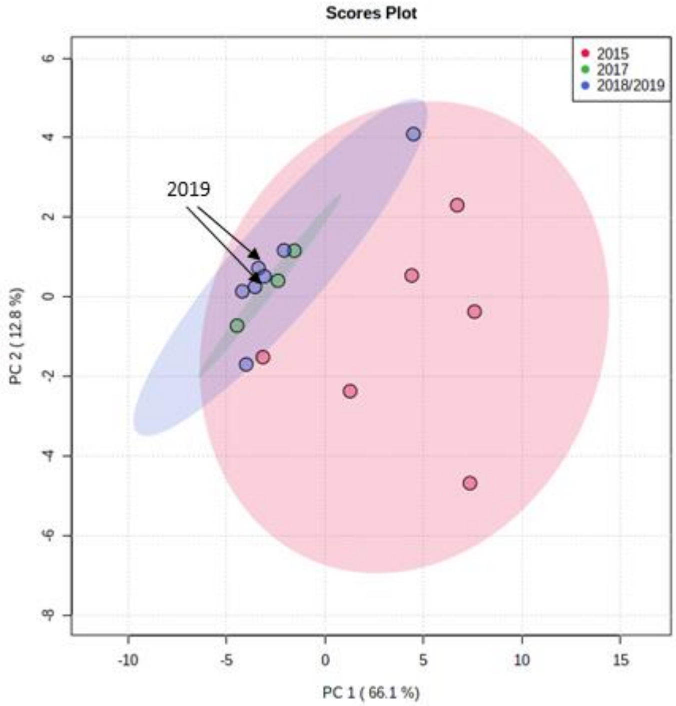

B

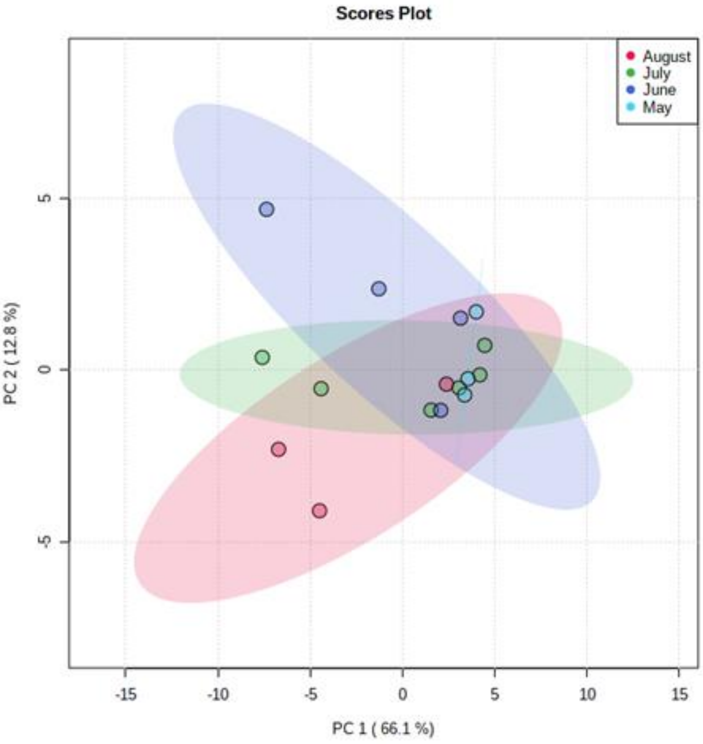

Supplementary Figure S4. Scores plots between the first and second principal components (with explained variances shown in brackets) obtained in principal component analysis (PCA) of amino acid profiles in honeybee venom. A – year of sample collection was taken as a grouping variable. At least three replicates are required in each group, therefore samples collected in 2018 (n=5) and 2019 (n=2) were combined in one group. B- month of sample collection was taken as a grouping variable. Before the PCA data were autoscaled.

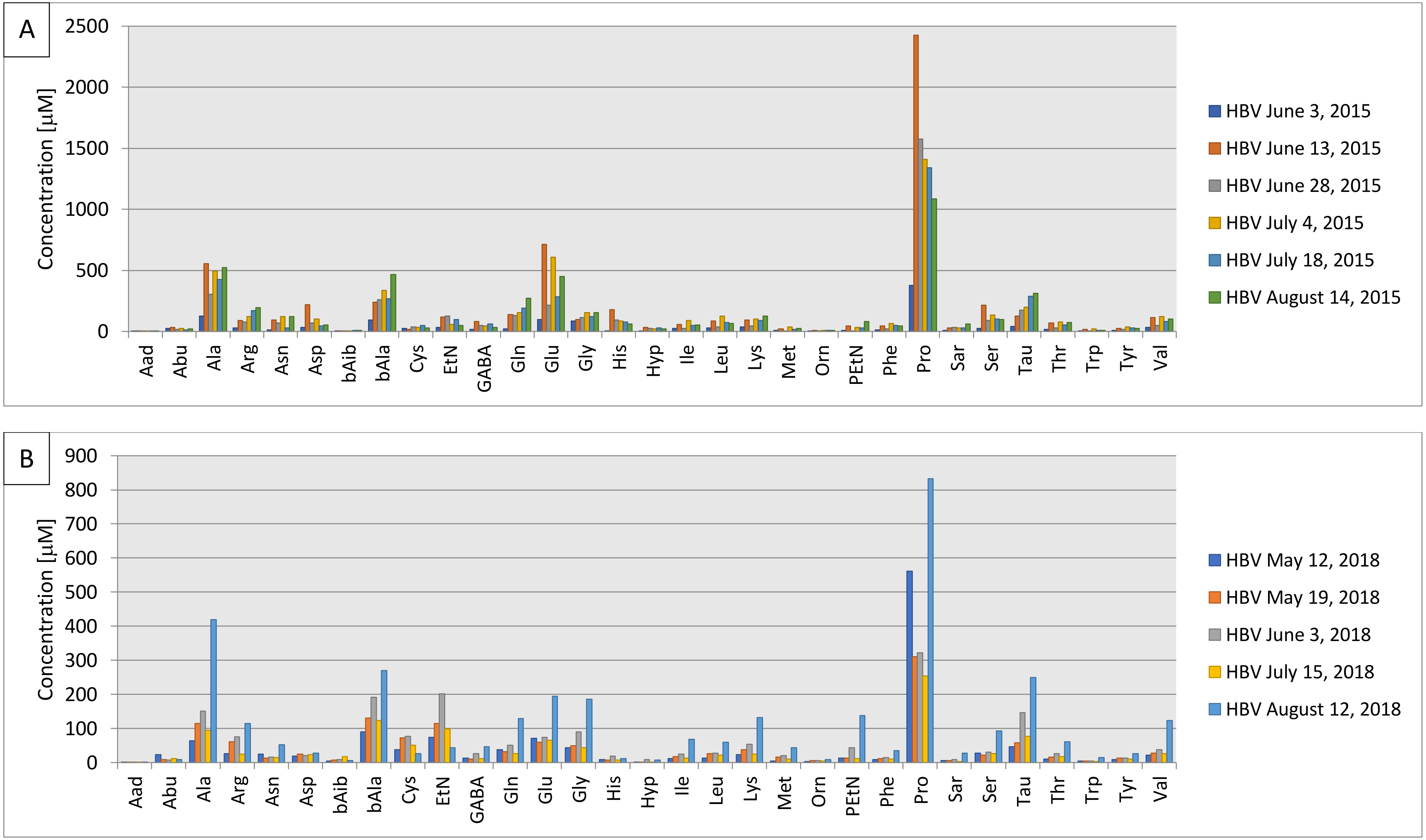

Supplementary Figure S5. Amino acid profiles determined in solutions of honeybee venom (c=50 mg/ml) collected in different months and years (A – 2015, B- 2018). Amino acid abbreviations are defined in Table S3.
